# Polylactide Composites Reinforced with Pre-Impregnated Natural Fibre and Continuous Cellulose Yarns for 3D Printing Applications

**DOI:** 10.3390/ma17225554

**Published:** 2024-11-14

**Authors:** Lakshmi Priya Muthe, Kim Pickering, Christian Gauss

**Affiliations:** School of Engineering, The University of Waikato, Hamilton 3216, New Zealand; lm242@students.waikato.ac.nz (L.P.M.); kim.pickering@waikato.ac.nz (K.P.)

**Keywords:** bio-derived composites, natural fibres, regenerated cellulose, additive manufacturing

## Abstract

Achieving high-performance 3D printing composite filaments requires addressing challenges related to fibre wetting and uniform fibre/polymer distribution. This study evaluates the effectiveness of solution (solvent-based) and emulsion (water-based) impregnation techniques to enhance fibre wetting in bleached flax yarns by polylactide (PLA). For the first time, continuous viscose yarn composites were also produced using both impregnation techniques. All the composites were carefully characterised throughout each stage of production. Initially, single yarns were impregnated and consolidated to optimise formulations and processing parameters. Solution impregnation resulted in the highest tensile strength (356 MPa) for PLA/bleached flax filaments, while emulsion impregnation yielded the highest tensile strength for PLA/viscose filaments (255 MPa) due to better fibre wetting and fibre distribution. Impregnated single yarns were then combined, with additional polymer added to produce filaments compatible with standard material extrusion 3D printers. Despite a reduction in the mechanical performance of the 3D-printed composites due to additional polymer impregnation, relatively high tensile and bending strengths were achieved, and the Charpy impact strength (>127 kJ/m^2^) for the viscose-based composite exceeded the reported values for bio-derived fibre reinforced composites. The robust mechanical performance of these filaments offers new opportunities for the large-scale additive manufacturing of structural components from bio-derived and renewable resources.

## 1. Introduction

The 3D printing industry is increasingly focused on sustainable material alternatives, with data showing that thermoplastics are the most commonly used materials and that material extrusion methods, especially FDM (fused deposition modelling), are the most prevalent technologies [1]. However, the heavy reliance on petrochemical-based thermoplastics conflicts with the goals of a circular economy, prompting a strong interest in bio-derived polymers and their composites to enhance sustainability [2].

Various bio-derived fibres, such as wood, hemp, bamboo, flax, harakeke, lyocell, and nanofibrillated cellulose, have been used as short/discontinuous fibre reinforcements for 3D printing [3,4,5]. However, the enhancement of mechanical properties using short/discontinuous fibres is usually limited due to limited load transfer, lack of fibre alignment, and poor interfacial adhesion [3]. The integration of long or continuous fibres has shown substantial potential for enhancing the mechanical properties of bio-derived composites, marking a significant step toward achieving high-performance 3D-printed composites [4,5,6,7]. Although fibres such as flax, ramie, and jute have been used to develop high-performance composites for FDM 3D printing, their mechanical properties still fall short of those achieved with traditional long/continuous fibre composites made through compression moulding techniques [3,8]. Factors like the twisted structure of flax yarn, a weak fibre-matrix interface, and limited interlayer adhesion inherent to the FDM process are primary contributors to the reduced mechanical strength of these composites [9].

Regenerated cellulose fibres such as viscose have only recently been studied as alternatives to twisted yarns as continuous reinforcements. These fibres have the advantages of high tensile strength, a bio-derived nature, and predictable mechanical behaviour, and are available commercially in the form of continuous fibres [10]. A recent study employing commercial viscose yarns as reinforcements for 3D printing demonstrated that the tensile strength and modulus of the composite increase by four and twenty times, respectively, compared to neat polymer [11]. Hence, the use of continuous regenerated cellulose fibres as a bioderived reinforcement could be of great benefit for 3D printing.

Polylactide (PLA)/flax composites with 44 wt% fibre are reported to have the highest tensile strength (293 MPa) and Young’s modulus (24.7 GPa) in the present reported literature for FDM composites [12]. In this study, flax yarns were pre-treated with silane coupling agents to improve compatibility with PLA, and an impregnation block was employed to infiltrate molten PLA into the yarns, producing filaments for 3D printing [12]. Recent research also highlights that untwisting the fibres can enhance the mechanical properties of long flax/PLA composites produced via FDM 3D printing [13].

Insufficient wetting and inadequate impregnation continue to be key challenges in improving the mechanical performance of these long/continuous natural fibre-reinforced composites. Moreover, the literature lacks extensive information on using impregnation methods to enhance interfacial bonding, a strategy that has shown success in compression moulding techniques. This study addresses these gaps by employing different polymer impregnation methodologies with high-strength flax and viscose fibre yarns to improve fibre wetting by PLA. The study compares a water-based emulsion with a solution/solvent-based impregnation process, optimises production through various impregnation formulations, and demonstrates a methodology to produce filaments reinforced with long/continuous fibres that can be used in FDM printers without major modifications.

## 2. Materials and Methods

### 2.1. Materials

**Figure 1:**
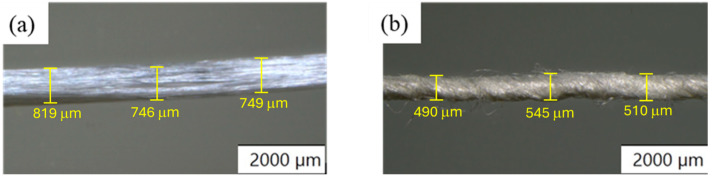
Stereomicroscope images of reinforcement yarns: (**a**) viscose, (**b**) bleached flax.

Viscose (regenerated cellulose) yarns, generously supplied by Cordenka GmbH (Obernburg, Germany), have a nominal yarn count of 2440 dTex (linear density of 4.1 Nm), with zero twist and a density of 1.5 g/cm^3^. Bleached flax yarns, obtained from Jos Vanneste (Harelbeke, Belgium), feature a yarn count of 2083 dTex (linear density of 4.8 Nm) and a density ranging from 1.3 to 1.4 g/cm^3^ [14]. Figure 1 presents stereomicroscope images of both viscose and bleached flax, and Table 1 provides a summary of the tensile properties of the individual fibres. PLA grade 2003D, with a specific gravity of 1.24 g/cm^3^ and a melt flow index of 6 g/10 min, was sourced from NatureWorks^®^ (Plymouth, MN, USA). Dichloromethane (DCM), used as a solvent, was supplied by Sigma Aldrich (Auckland, New Zealand). Additionally, PL1005-grade emulsion, containing 40 wt% PLA/water with a nominal particle size of 5 μm, was provided by Miyoshi Oil & Fat Co., Ltd. (Tokyo, Japan). The methodology for the tensile testing of single fibres and the additional characterisation results of the fibres and PLA grades used in this study are available in the Supplementary File.

### 2.2. Filament Production Methodology

#### 2.2.1. Impregnation and Consolidation of Single Filament

**Figure 2:**
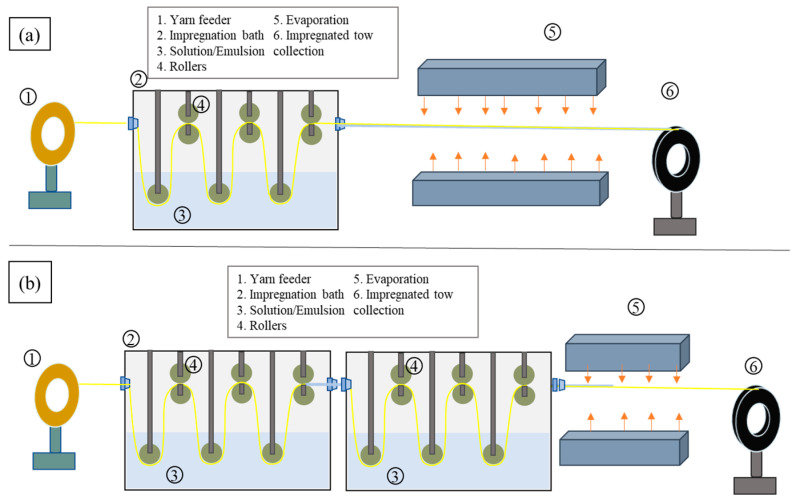
Impregnation process with (**a**) a single impregnation bath and (**b**) two baths in tandem.

A schematic of the impregnation process is shown in Figure 2. Initially, a single impregnation bath (Figure 2a) was used, which was later updated to have two baths in tandem, as shown in Figure 2b. The solution impregnation method involves vacuum drying PLA (45 °C for 4 h) and yarns (100 °C for 24 h), followed by preparing 10 wt% and 7 wt% PLA solutions in DCM. Yarns are then fed through the impregnation bath containing the solution. Wing nuts were used to adjust the squeezing rollers in the impregnation bath, allowing the gap to be precisely set for the impregnated yarn to pass through. Agitation was maintained with a magnetic stirrer, while excess resin was removed via a nozzle with a 1 mm diameter. The impregnated tow undergoes solvent evaporation before collection onto a winding mandrel.

The emulsion impregnation process employs the same setup but requires drawing the yarns through a water-based PLA emulsion in the impregnation bath, where micro-PLA particles adhere to the fibre surfaces. Excess emulsion is removed in a similar manner, and the impregnated yarn is then dried before being collected. The key difference lies in the use of a PLA solution versus emulsion for impregnation, with the former involving a dissolved PLA solution and the latter utilising PLA emulsion for impregnating yarns. The formulations used for solution and emulsion impregnation processes are summarised in Table 2.

**Figure 3:**
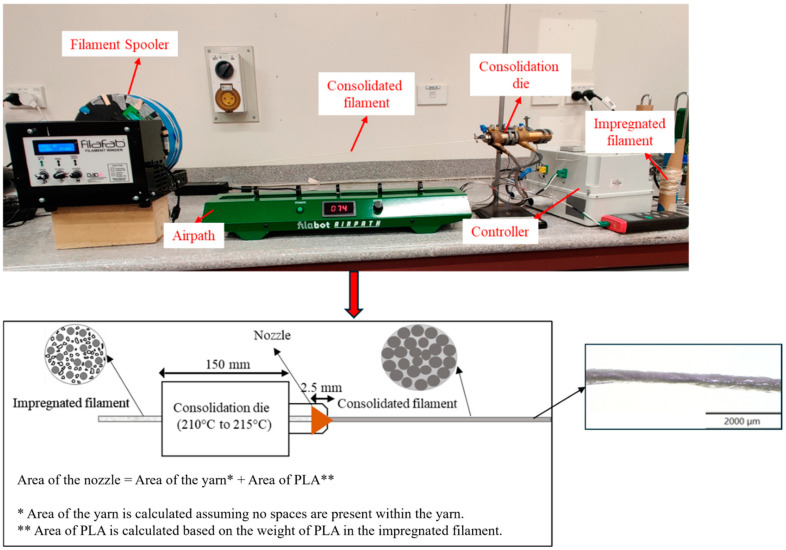
Consolidation process of impregnated filaments.

To improve matrix-to-fibre bonding and eliminate voids, the impregnated yarns underwent a consolidation process. The impregnated filament was dried in a vacuum oven at 80 °C for six hours, then passed through a heated consolidation die. Figure 3 provides the die dimensions and the calculation for the exit nozzle area. The die’s entrance and exit were tapered to prevent void formation, with the die temperature maintained between 210 °C and 215 °C.

#### 2.2.2. Production of 3D Printing Filament

**Figure 4:**
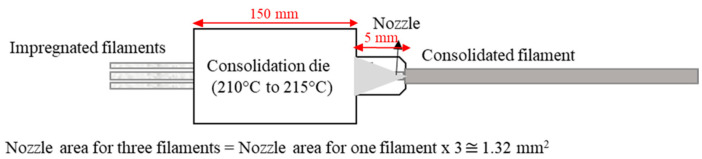
Consolidation process of three filaments into one filament.

Printing with a single consolidated filament was not feasible on a commercial 3D printer, so multiple filaments were combined, and additional polymer was incorporated via melt impregnation. This method produced an average filament diameter of 1.45 ± 0.1 mm, ensuring compatibility with commercial 3D printers. The impregnation formulations that yielded the highest tensile properties were selected to produce 3D printing filaments. PLA 2003D granules were used to impregnate additional polymer into these filaments. Initially, three individual filaments were combined into a larger filament using the consolidation method illustrated in Figure 4. Commercial Filabot extruder nozzles (Barre, VT, USA) with modified diameters of 1.45 mm were utilised for consolidation. These nozzles were chosen for their extended tapered section, unlike those previously used, allowing for a longer consolidation time to minimise porosity [13].

**Figure 5:**
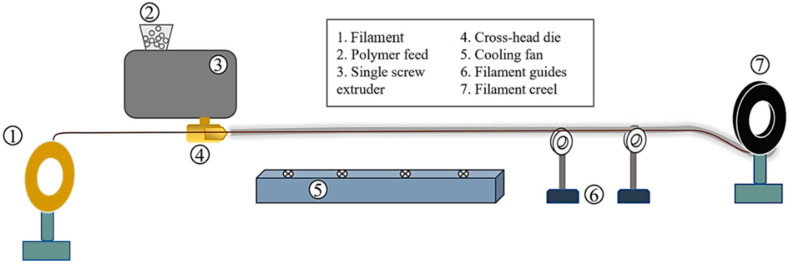
Production of 3D printing filaments via melt-impregnation of pre-consolidated filaments.

The consolidated filament, created by combining three filaments into one, had a high fibre content (60% to 70%), which caused nozzle clogging and print failures. To address this, an additional melt impregnation step was added to optimise both the filament diameter and fibre content. Figure 5 shows the production setup where the combined filament is fed into a crosshead die attached to a Filabot single-screw extruder (Barre, VT, USA). The crosshead die was maintained at 190 °C, allowing molten PLA to be injected continuously, impregnating the filament with polymer. The filament then passes through a cooler (Filabot Airpath, Barre, VT, USA), where it is cooled before being wound onto a creel using a spooling system. Extrusion and spooling speeds were carefully controlled to maintain a consistent filament diameter of 1.45 ± 0.5 mm.

### 2.3. Characterisation of the Filaments

#### 2.3.1. Optical Microscopy

Cross-sectional images of all composite filaments were captured with an Olympus BX53 optical microscope (Tokyo, Japan). Samples were prepared by mounting the samples in epoxy resin. After curing for 24 h at room temperature, the samples were removed, ground, and polished to expose the filament cross-sections. Grinding was performed with a sequence of abrasive papers (320, 500, 1000, 2000, and 4000 grit), followed by polishing with a Tegramin-25 auto-polishing machine using an OP-U oxide polishing suspension provided by Struers (Rodovre, Denmark).

#### 2.3.2. Porosity and Reinforcement Weight Percentage Analysis

The porosity of the composite filaments at each production stage was estimated according to the ASTM D792-20 standard [15]. This method calculates porosity by comparing the experimental density of the composite filament with its theoretical density. The experimental density is determined using Archimedes’ principle, as shown in Equation (1) [16].

(1) $$\rho_{ce}=\frac{M_{a}}{\left(M_{a}-M_{w}\right)}\times \rho_{w}$$

$\rho_{ce}$ is the experimental density of the composite filament sample. $M_{a}$ is the mass of the composite filament in air, $M_{w}$ is the mass of the composite filament in water, and $\rho_{w}$ is the density of water. Theoretical calculation of the density of the composite filament can be conducted using the rule of mixtures given by Equation (2) [16].

(2) $$\rho_{ct}=\rho_{f}V_{f}+\rho_{m}V_{m}$$

$\rho_{ct}$ is the theoretical density of the composite sample. $\rho_{f},\rho_{m}$ are the densities of fibre and matrix, respectively, and $V_{f},V_{m}$ are the volume fractions of fibre and matrix, respectively. Theoretical densities for the PLA matrix (1.24 g/cm^3^) and viscose fibres (1.5 g/cm^3^) were supplied by the manufacturer, while the density for flax fibres (1.4 g/cm^3^) was sourced from the literature [14]. By comparing these theoretical density values with the experimentally measured density of the composite, the porosity percentage can be calculated using Equation (3) [17].

(3) $$\%\;porosity=\{(\rho_{ct}-\rho_{ce})\;÷\;\rho_{ct}\}\times 100\%$$

A weight analysis was performed to determine the PLA content in the yarns. Five 1-m sections were selected for each reinforcement and impregnation technique. Each section was weighed both before and after impregnation, and these measurements, along with the yarns’ linear density, were used to calculate the reinforcement percentage.

#### 2.3.3. Tensile Properties of Composite Filaments

The tensile properties of the composite filaments were assessed throughout each stage of production. Experimental tensile testing was performed by mounting the composite filaments into 3D-printed tabs, which were designed with a groove matching the filament’s diameter and printed using commercial PLA filament. The composite filament was placed in the groove, and epoxy was applied to bond the two tabs together with the filament centred inside. Adhesive tape held the tabs in place until the epoxy cured. Specimens were cured under 0.5 tonnes of pressure in a manual hydraulic press at room temperature for 24 h. After curing, samples were conditioned in a climatic chamber (Binder GmbH, Model KMF 115, Tuttlingen, DE, USA) for 48 h at 23 °C and 50% relative humidity before testing.

Testing was conducted using an Instron^®^ 5982 universal testing machine (Norwood, MA, USA) with a 500 N load cell. Filament diameters were measured with an Olympus BX60F5 optical microscope at five different points, and the average diameter was used for calculations. Testing was performed at a crosshead displacement rate of 5 mm/min with a gauge length of 30 ± 2 mm. Due to the small diameter of single-impregnated filaments, an extensometer was not used. Instead, a mild steel block with known mechanical properties was used as a reference material to correct for displacement errors. For consolidated and 3D printing filaments, tensile strain was measured with a 10 mm clip-on extensometer. Five specimens were tested for each composite type.

#### 2.3.4. Scanning Electron Microscopy Analysis of Cryofracture Surfaces and Tensile Fracture Surfaces

A Hitachi S-4000 field emission scanning electron microscope (Tokyo, Japan), operated at 3 kV, was used to examine the cryofracture surfaces and fracture behaviour of the composite filaments. Samples were mounted on aluminium stubs with carbon tape and sputter-coated with platinum prior to analysis.

### 2.4. FDM 3D Printing

**Figure 6:**
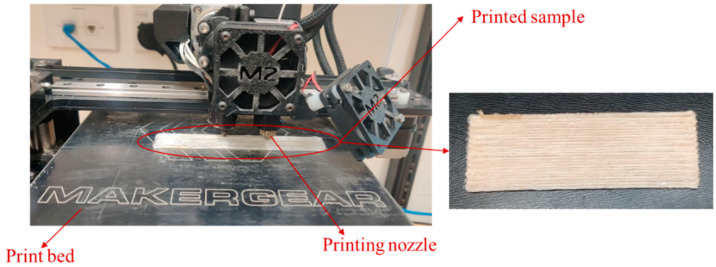
FDM 3D printing of long/continuous bio-derived fibre reinforced PLA composite.

A MakerGear^®^ M2 desktop printer was used to produce samples for tensile (Beachwood, OH, USA), flexure, and impact testing. Samples were cut from 3D-printed rectangular shapes to meet the required dimensions for mechanical testing. Figure 6 shows the 3D printing process in progress. All samples were printed with 100% infill density, using a 1.5 mm nozzle, a raster angle of 0°, and a layer height of 0.8 mm. The bed temperature was set at 60 °C, the nozzle temperature at 190 °C, and the printing speed at 300 mm/min. Upon completion of printing, the filament was manually cut to prevent any movement or displacement of the printed sample. Control samples of pure PLA were printed with the same settings to allow for a consistent comparison with the composite samples.

### 2.5. Characterisation of the 3D-Printed Composite

#### 2.5.1. Mechanical Characterisation

**Figure 7:**
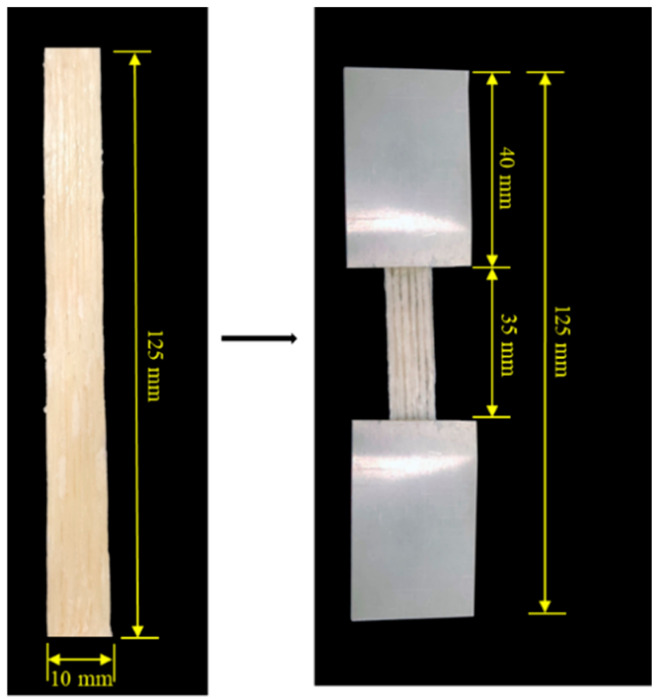
A 3D-printed tensile specimen (**left**) and a mounted specimen for tensile testing (**right**).

The 3D-printed specimens were tested along the fibre direction with end tabs in accordance with ISO 527-5 standard [18]. Tensile testing was conducted on an Instron^®^ 5982 tensile tester equipped with a 5 kN load cell. Aluminium end tabs were attached to the specimens using Araldite epoxy, as shown in Figure 7. Testing was performed with a crosshead displacement rate of 5 mm/min, and tensile strain was measured using a 10 mm clip-on extensometer. Five specimens were tested for each composite type.

Three-point bending tests on the 3D-printed composites were conducted using an Instron^®^ 5982 mechanical testing machine with a 5 kN load cell. Samples measuring 85 × 15 × 2.5 mm were tested over a span length of 55 mm, with five replicates for each composite type, following ASTM D790-17 standard [19]. Prior to testing, samples were conditioned in a climatic chamber at 23 °C and 50% relative humidity for 48 h.

The Charpy impact strength of the 3D-printed composites was evaluated in accordance with ISO 179-1 standard [20]. A Ray-Ran universal pendulum impact tester (Warwick, UK) with a 1.19 kg hammer and a test speed of 2.9 m/s was used. For each condition, five unnotched, rectangular specimens (80 × 10 × 4 mm) were tested.

#### 2.5.2. SEM of Fracture Surfaces

A Hitachi S-4000 field emission scanning electron microscope, operating at 3 kV, was employed to examine the fracture surfaces of the 3D-printed specimens from the tensile tests. Samples were mounted on aluminium stubs using carbon tape and then sputter-coated with platinum prior to observation.

## 3. Results and Discussion

### 3.1. Single Yarn-Reinforced Filaments

**Figure 8:**
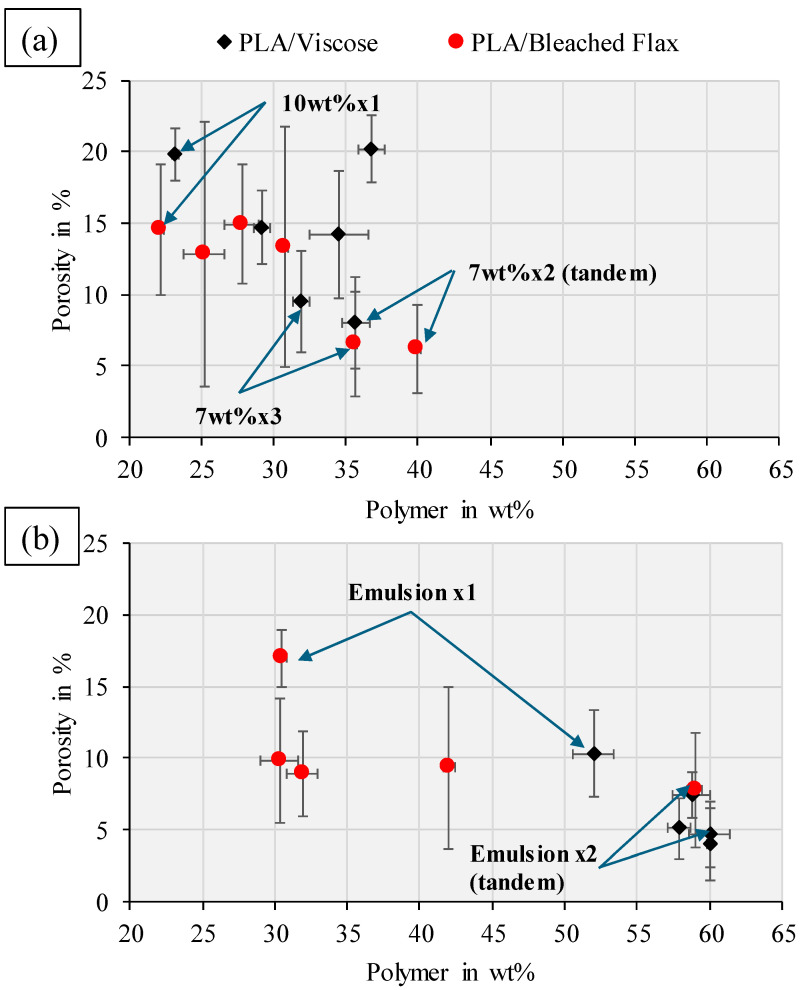
Porosity vs. polymer wt% for different viscose and bleached flax-based single yarn-reinforced filaments produced by (**a**) solution impregnation; (**b**) emulsion impregnation. The formulations with the lowest and highest porosity percentages are indicated using the blue arrows.

Figure 8 presents the polymer weight percentages and total porosities for all single yarn-reinforced filament formulations produced through solution and emulsion impregnation, followed by consolidation (with detailed data available in Tables S3 and S4). The overall trend showed that as the polymer content increased, the porosity percentage generally decreased, which aligns with findings reported in other studies. Reports in the literature have also documented increased porosity levels (exceeding 6.8%) in flax yarn-reinforced composites with high fibre content (>50 wt%) [21,22]. The significant variation in porosity values, particularly in the PLA/bleached flax composites, is attributed to the heterogeneous impregnation of the twisted yarn and possibly insufficient pressure during the impregnation process. In contrast, the untwisted viscose yarn exhibits much lower variation in porosity.

The 40 wt% PLA emulsion impregnated more PLA into the yarns than the 10 wt% or 7 wt% DCM solutions. Water’s higher polarity, compared to DCM, enables stronger hydrogen bonding with the cellulose hydroxyl groups, leading to increased fibre absorption and swelling. In contrast, DCM’s lower polarity results in less fibre affinity and absorption. Consequently, the wetting capability of water surpasses that of DCM, potentially increasing PLA particle deposition on fibre surfaces when using the PLA/water emulsion compared to the PLA/DCM solution. However, the twist in flax yarns complicates the impregnation process. While PLA/bleached flax filaments achieved higher polymer content, it is crucial for the polymer to penetrate the yarn twist and uniformly wet the fibres. Reducing the PLA/DCM solution viscosity to 7 wt% was intended to improve polymer penetration and fibre wetting at the yarn’s centre. Using two impregnation cycles with the 7 wt% PLA/DCM solution enhanced central fibre wetting for PLA/bleached flax, though some voids remained.

**Figure 9:**
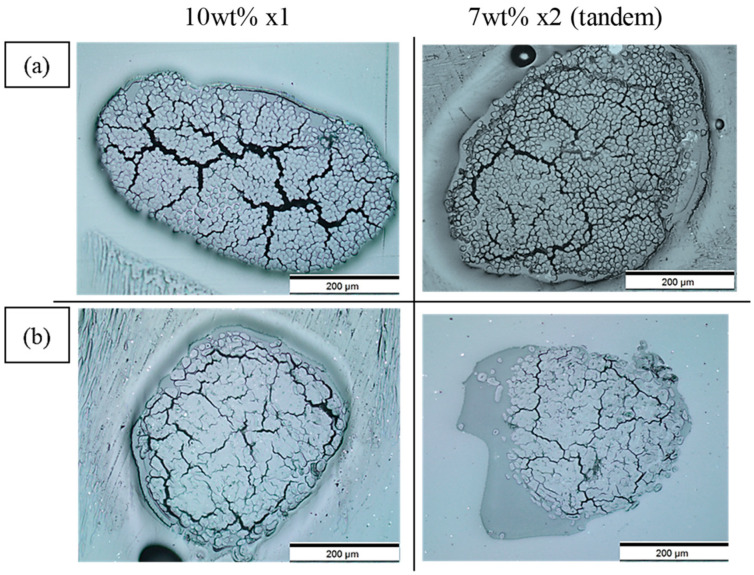
Optical microscopy images of cross sections of solution-impregnated filaments for 10 wt% (**left**) and 7 wt% x2 (tandem) (**right**) formulations with (**a**) PLA/viscose; (**b**) PLA/ bleached flax.

**Figure 10:**
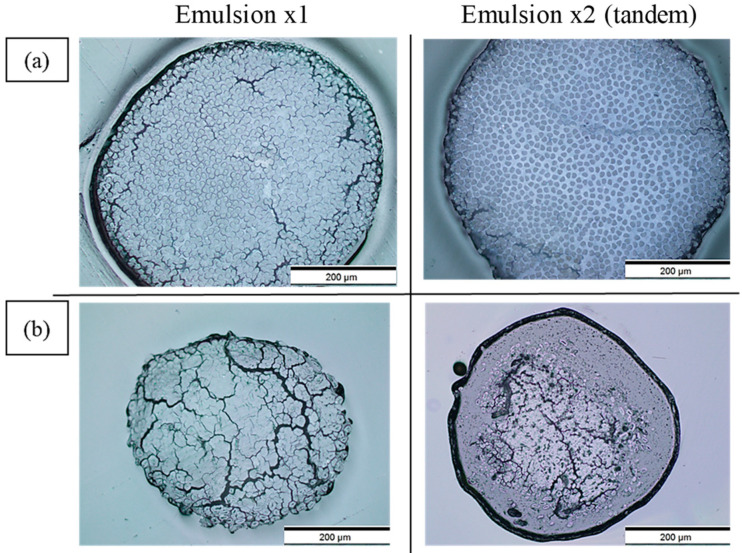
Optical microscopy images of cross sections of emulsion-impregnated filaments for Emulsion x1 (**left**) and Emulsion x2 (tandem) (**right**) formulations with (**a**) PLA/viscose; (**b**) PLA/bleached flax.

**Figure 11:**
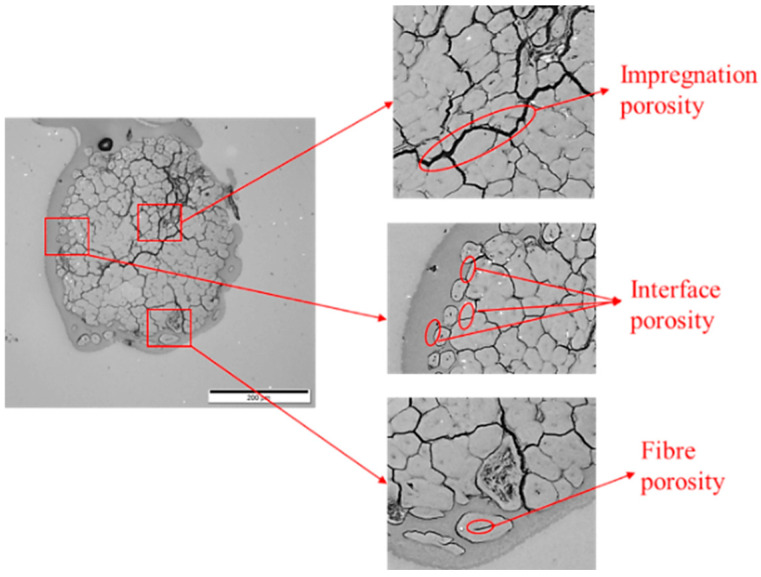
Types of porosities observed in optical microscope images of consolidated filaments.

Figure 9 and Figure 10 show the cross-sectional views of the filaments obtained via optical microscopy, with images for all formulations provided in the Supplementary File (Figures S14–S17). These images reveal voids with a crack-like structure, along with other smaller voids. As illustrated in Figure 11, the voids are categorised into impregnation, interface, and fibre porosity types [23]. Impregnation porosity, observed as crack-like voids, is present in all filaments, suggesting incomplete polymer impregnation. Interface porosity, due to weak interfacial adhesion, is more pronounced in PLA/viscose filaments, likely because of the smooth surface of viscose fibres (refer to Figure S5). Fibre porosity, originating from the lumen in bio-derived fibres, is only found in filaments reinforced with bleached flax.

**Figure 12:**
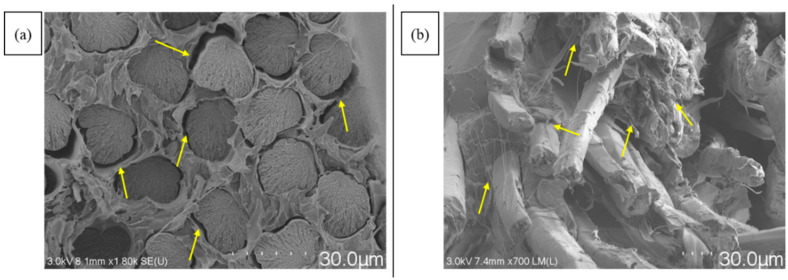
SEM images of cryofracture surfaces depicting mechanical interlocking between PLA and fibres—(**a**) PLA/viscose and (**b**) PLA/bleached flax (scale bar = 30 µm). The yellow arrows indicate the gaps between fibre and matrix after cryofracture.

PLA/viscose filaments exhibited homogeneous polymer and fibre distribution, especially after two cycles using the emulsion impregnation method, as evidenced in Figure 10a. PLA/bleached flax, on the other hand, displayed matrix pooling at edges and low polymer content in the centre, possibly due to the twisted nature of flax yarn. Mechanical interlocking was higher in bleached flax filaments, potentially due to rougher fibre surfaces creating sites for polymer adhesion, as highlighted by the yellow arrows in cryofracture surfaces shown in Figure 12 [24,25,26,27]. Improvement in tensile properties and an increase in crystallinity due to surface roughness have been reported with the use of bleached fibres in multiple studies [28,29]. Cryofracture surface images for all the formulations are provided in the Supplementary File (Figures S10–S13).

**Figure 13:**
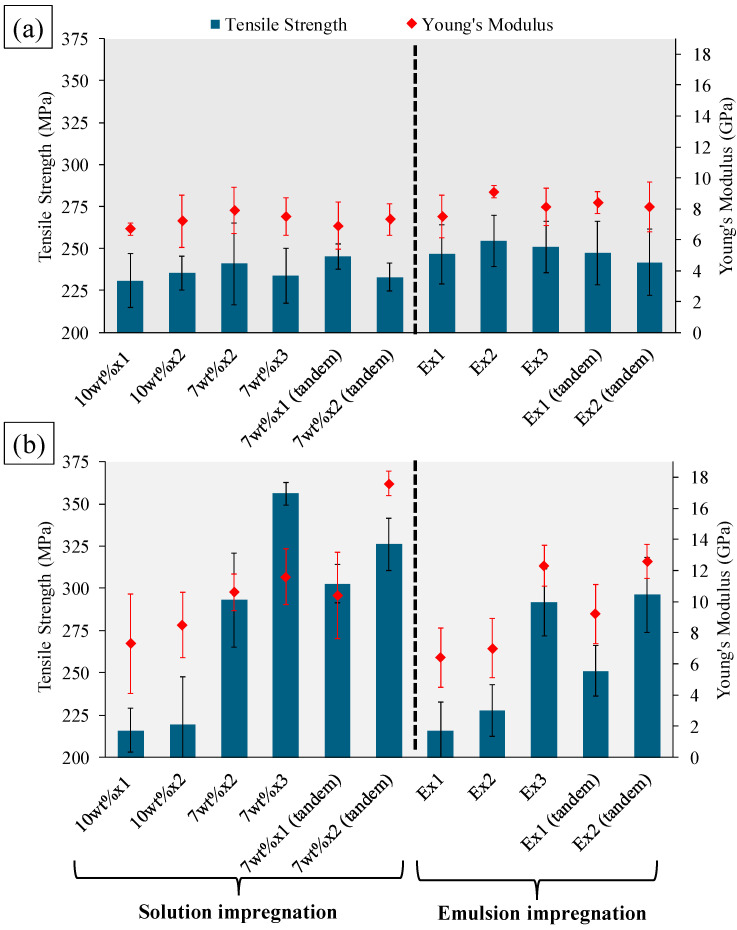
Tensile strength and Young’s modulus of single yarn-reinforced filaments: (**a**) PLA/viscose, (**b**) PLA/bleached flax.

The tensile properties of single yarn composite filaments produced from solution and emulsion impregnation processes are shown in Figure 13. The complete tensile test data, along with the strain at break percentages, are given in Tables S5 and S6 of the Supplementary File. For PLA/viscose composites, an optimum polymer content of 58.8 wt% for the Emulsion x2 formulation resulted in the highest tensile strength of 254.7 ± 15.3 MPa and a Young’s modulus of 9.1 ± 0.4 GPa. In the case of PLA/bleached flax composites, formulations with lower porosity resulted in higher tensile properties. The 7 wt% x3 solution impregnation formulation with PLA/bleached flax exhibited the highest tensile strength (356.1 ± 6.8 MPa), while the 7 wt% x2 (tandem) had the highest Young’s modulus (17.6 ± 0.8 GPa).

**Figure 14:**
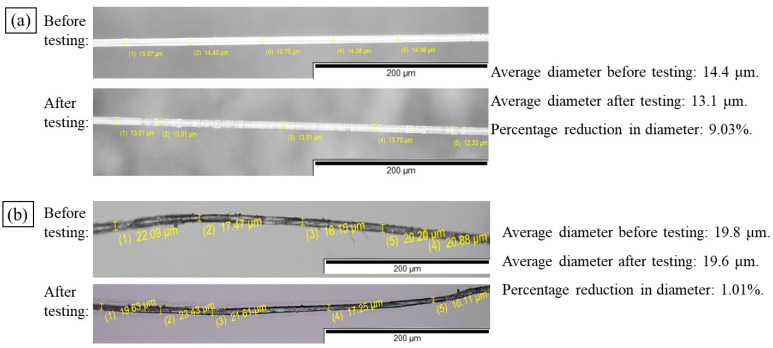
Analysis of percentage reduction in viscose (**a**) and bleached flax fibre diameters (**b**).

Notably, the strain at break for PLA/viscose filaments was significantly higher than that of individual viscose fibres (εb of 10.7–15.6% for the composites compared to a εb of 13.2% for single fibres). This difference may be attributed to fibre sliding within the filament, caused by reduced fibre diameter during tensile testing. Viscose fibres generally exhibit a greater reduction in diameter than flax fibres under tensile stress, which can lead to fibre separation and sliding, resulting in increased strain at break values. Figure 14 illustrates the diameter differences for individual viscose and bleached flax fibres before and after tensile testing, highlighting the reduction observed in viscose fibres.

While multiple studies have investigated the properties of 3D-printed composites, filament characterisation has not been extensively covered, with only two studies reporting on long/continuous bio-derived fibre-reinforced composite filaments [30]. In this study, the highest tensile strength achieved (356.1 ± 6.8 MPa at 64.3 wt% fibre content for PLA/bleached flax) represents the strongest tensile strength for PLA/flax composites reported in the literature to date. For comparison, the highest previously reported tensile strength for PLA/flax composites was 339.0 ± 22 MPa, obtained using compression moulding techniques [31]. Additionally, the highest Young’s modulus in this study was observed in the 7 wt% tandem impregnation formulation for PLA/bleached flax composites, reaching 17.6 ± 0.8 GPa. Higher Young’s modulus values have been noted in other studies, albeit with lower reinforcement percentages than used here [12]. The reduction in Young’s modulus could be due to the dominant twist in the flax yarns used for this work. The linear density of bleached flax yarns used in the present study is 2083 dtex, corresponding to an approximate twist angle of 17°. The twist angle was calculated according to a mathematical model presented by Darshil et al., which correlates yarn linear density (tex) and yarn structure. The study also documented a reduction of approximately 50% in the tensile properties of flax yarn-reinforced composites when the yarn was twisted to an angle of 17°, compared to untwisted yarn. This finding is consistent with the results observed in the present study [32].

**Figure 15:**
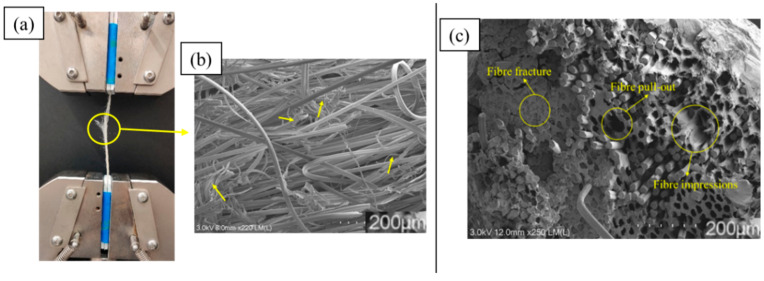
(**a**) Macroscopic failure of tensile-tested solution-impregnated PLA/viscose composite filaments; (**b**) SEM images of fracture surface of solution-impregnated PLA/viscose composite filament; (**c**) SEM image of fracture surface of emulsion-impregnated PLA/viscose composite filaments (scale bar = 200 µm) (Yellow arrows in (**b**) indicate the areas of polymer debonding).

Solution-impregnated PLA/viscose composites exhibited a brush-like failure mode, as shown in Figure 15a. The failure surface displayed a mix of fibre debonding and pull-out. Areas of polymer debonding, along with polymer strings, are marked by yellow arrows in the SEM images in Figure 15b. An SEM image of the fracture surfaces of PLA/viscose filaments produced with the emulsion impregnation technique is shown in Figure 15c. The fracture surfaces revealed that some viscose fibres remained embedded in the matrix, indicating effective stress transfer, while others were pulled out. Brittle fracture surfaces of the viscose fibres were also visible, along with regions where fibre impressions were left on the matrix. These features suggest that there is potential for the further enhancement of interfacial bonding [33,34].

**Figure 16:**
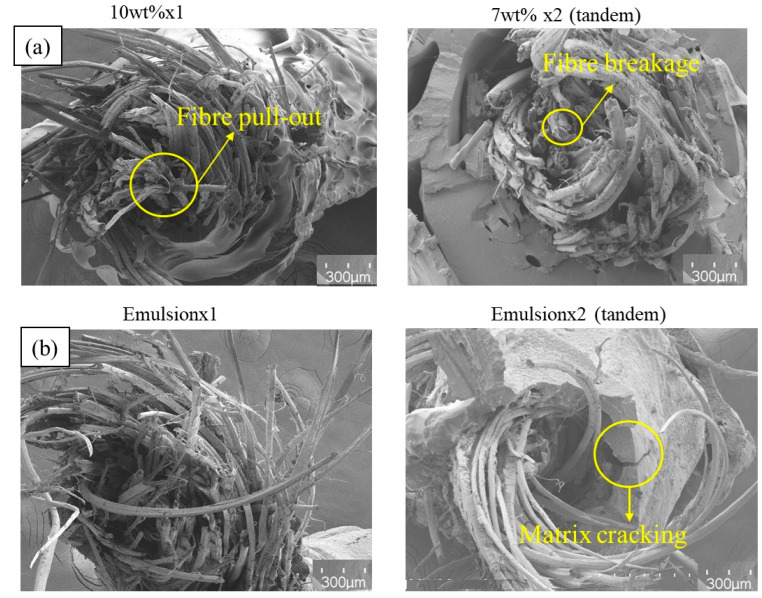
SEM images of fracture surfaces of bleached flax filaments: (**a**) solution impregnation, (**b**) emulsion impregnation (scale bar = 300 µm).

The failure surfaces of the PLA/bleached flax composites are shown in Figure 16a for the solution impregnation method and Figure 16b for the emulsion impregnation method. Both impregnation techniques exhibited similar fracture behaviours, characterised by significant fibre pull-out due to debonding between the fibre and matrix [35,36]. In high-performing flax fibre-reinforced composites, fewer fibre pull-outs and more fibre breakage were observed, indicating stronger interfacial bonding between the fibres and the matrix [37]. Emulsion-impregnated flax filaments also showed matrix cracking, alongside fibre pull-outs and debonding, as highlighted in Figure 16. Matrix cracking is often associated with the presence of voids or inclusions within the matrix [38].

Based on the results of the single-yarn composites, only the formulations with the best tensile properties were used for the production of 3D printing filaments. A summary of the tensile properties of these formulations is given in Table 3.

### 3.2. Multiple Consolidated and 3D Printing Filaments

**Figure 17:**
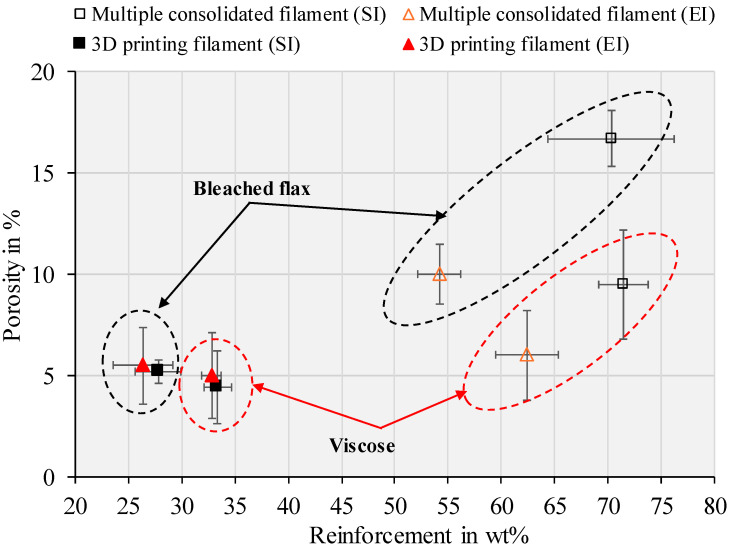
Porosity % vs. reinforcement wt% of viscose and bleached flax-based multiple consolidated and 3D printing filaments (SI—solution impregnation; EI—emulsion impregnation).

The fibre weight and porosity percentages for the multiple consolidated and 3D-printed filaments, based on the formulations in Table 3, are plotted in Figure 17 (with detailed data in Tables S7 and S8). Consolidating three filaments into one resulted in a slight increase in fibre weight percentage across all composites, compared to the single filaments. Specifically, solution- and emulsion-impregnated viscose-reinforced composites had fibre contents of 71.4 and 62.4 wt%, respectively, while bleached flax-reinforced composites had fibre contents of 70.3 and 54.2 wt%. This increase in fibre content may be due to the application of heat and pressure during consolidation, which can lead to the partial removal of polymer from the composite structure into the consolidation die. A similar trend of fibre weight percentage increasing by 1% to 10% during consolidation has been observed in processes such as pultrusion and compression moulding [39,40]. However, the influence of an increased fibre weight percentage on mechanical properties depends on the uniformity of fibre distribution and porosity [41]. Following melt impregnation to increase filament diameter, all 3D-printed filaments exhibited lower porosity, ranging from 4.5% to 5.5%. This reduction in porosity is attributed to the denser matrix, which decreases the overall porosity of the filament. Additionally, the application of heat and pressure during melt impregnation may expel trapped air or moisture, contributing to the removal of voids [42].

**Figure 18:**
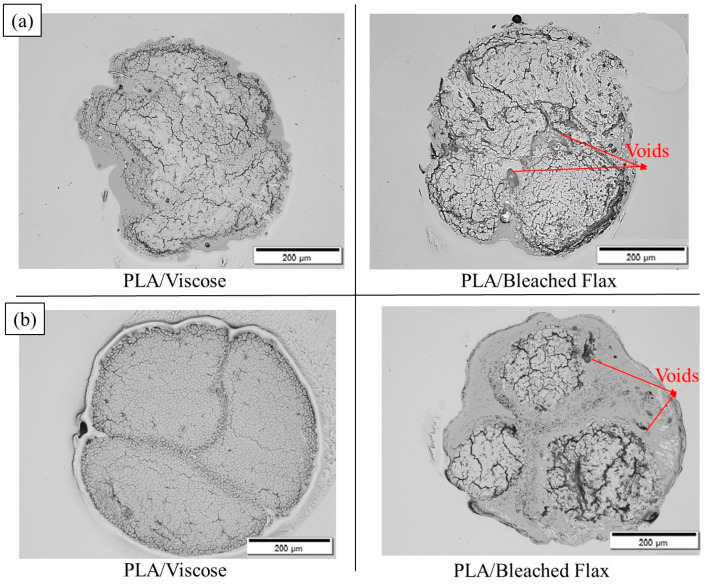
Optical microscope images of cross-sections of multiple consolidated filaments: (**a**) solution impregnation; (**b**) emulsion impregnation.

**Figure 19:**
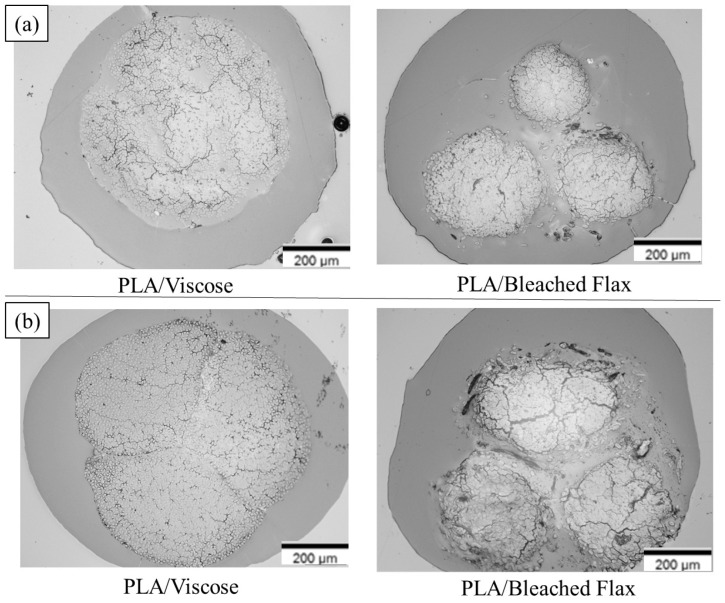
Optical microscope images of cross-sections of 3D printing filaments: (**a**) solution impregnation; (**b**) emulsion impregnation.

Figure 18 and Figure 19 display cross-sectional images of the multiple consolidated filaments and 3D printing filaments produced using both solution and emulsion impregnation techniques. Solution-impregnated PLA/viscose and PLA/bleached flax filaments showed good adhesion between individual filaments, with minimal void presence. In contrast, the emulsion-impregnated filaments exhibited fusion primarily at the boundaries of the individual filaments without full merging, unlike the solution-impregnated filaments, which demonstrated complete filament integration. This partial fusion in emulsion-impregnated filaments is likely due to the sintering effect from the emulsion particles, which reduces polymer viscosity and melt flow rate [43]. The untwisted viscose yarns used in this study were advantageous for the PLA/viscose filaments, resulting in a relatively uniform fibre distribution within the composite. However, the PLA/bleached flax filaments contained a few irregular, translucent voids, as shown in Figure 18, likely due to insufficient drying or trapped moisture [42].

The melt impregnation process appeared to add additional polymer around the filaments, but it did not enhance fibre distribution within them, possibly due to the high viscosity of the PLA matrix and insufficient pressure during impregnation. Nonetheless, the added polymer from melt impregnation could improve interlayer adhesion during 3D printing [4,44].

**Figure 20:**
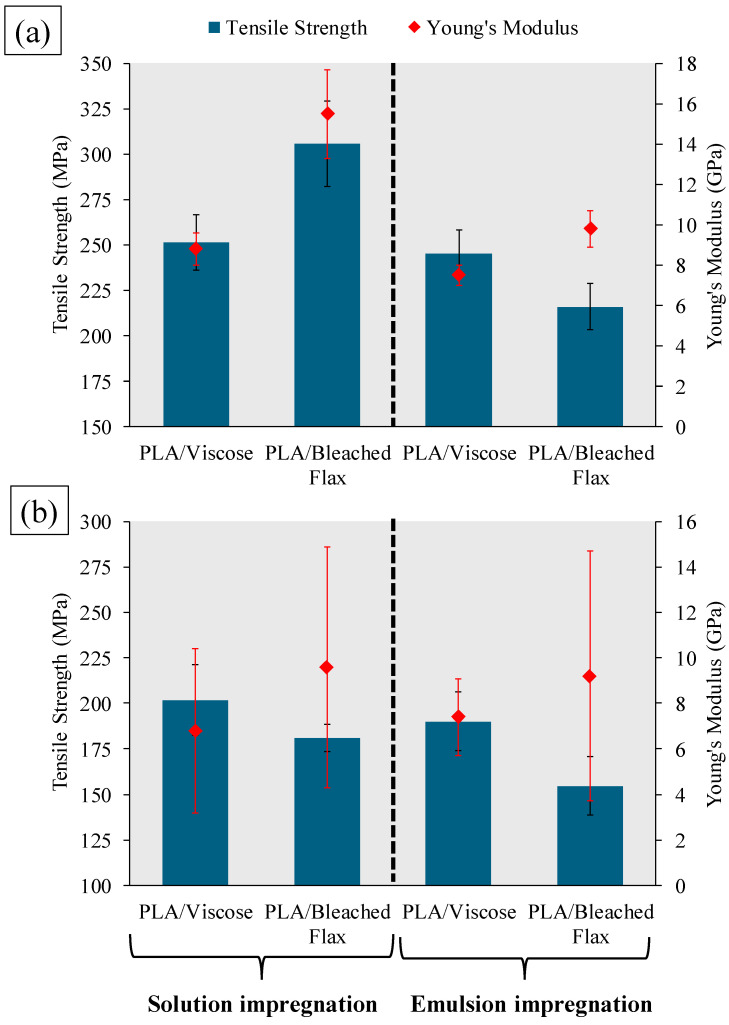
Tensile strength and Young’s modulus of (**a**) multiple consolidated (**b**) 3D printing filaments.

Figure 20 summarises the tensile properties of the multiple consolidated and 3D-printed filaments. Solution-impregnated PLA/viscose filaments demonstrated slightly higher tensile strength (251.2 ± 15.2 MPa) and Young’s modulus (8.8 ± 0.8 GPa) than single filaments, showing increases of approximately 4.3% and 11.4%, respectively. These results align with the microscopic and porosity analyses, which indicated increased fibre content without additional porosity in the PLA/viscose composites. Conversely, PLA/bleached flax composites showed reduced tensile strength (305.7 ± 23.8 MPa) and Young’s modulus (15.5 ± 2.2 GPa), decreasing by roughly 6.3% and 11.9%, likely due to poor adhesion between individual filaments and an increase in porosity-related defects [45].

For emulsion impregnation, all multiple consolidated filaments displayed reduced tensile properties compared to single filaments. Although the PLA/viscose filaments had slightly lower porosity in the consolidated form, a decrease in tensile strength (245.3 ± 13 MPa) and Young’s modulus (7.5 ± 0.5 GPa), with reductions of around 3.7% and 17.6%, respectively, was observed. This reduction may result from a decrease in PLA’s molecular weight caused by repeated melt processing [46,47,48]. PLA/bleached flax composites also exhibited declines in tensile strength (215.9 ± 12.8 MPa) and Young’s modulus (9.8 ± 0.9 GPa), with reductions of approximately 27.1% and 22.2%.

Adding PLA through melt impregnation further reduced the tensile properties of all composites due to increased matrix content. PLA/viscose filaments showed a lesser reduction in tensile properties than PLA/bleached flax, likely due to the differences in fibre content. After melt impregnation, PLA/viscose filaments averaged 33.4 wt% fibre content, while PLA/bleached flax filaments averaged 27.8%, leading to a more pronounced decrease in tensile properties for the latter.

**Figure 21:**
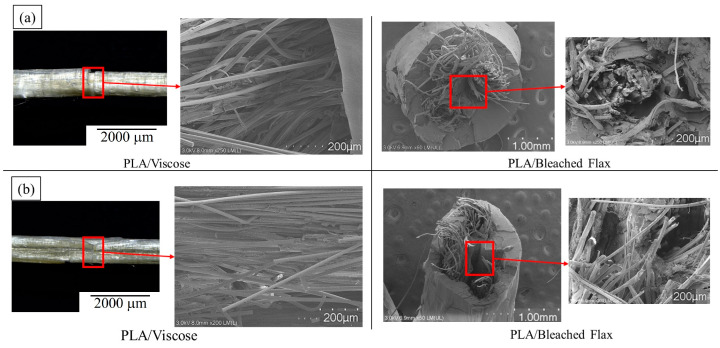
SEM images of fracture surfaces of 3D printing filaments: (**a**) solution impregnation; (**b**) emulsion impregnation.

Figure 21a,b display the macroscopic failure surfaces of PLA/viscose filaments and the SEM images of fracture surfaces for tensile-tested 3D printing filaments with solution and emulsion impregnation. The macroscopic failure surfaces of solution and emulsion-impregnated PLA/viscose filaments show polymer separation from fibres, suggesting weak adhesion between melt-impregnated PLA and viscose fibres. SEM images reveal debonding, fibre breakage, and fibre pullouts associated with interface porosity in both solution- and emulsion-impregnated PLA/viscose filaments [49]. In contrast, PLA/bleached flax filaments showed a combination of fibre pull-outs and a brittle fracture of the matrix in both solution- and emulsion-impregnated filaments, indicating poor impregnation and interfacial adhesion [4,50].

### 3.3. Fused Deposition Modelling 3D Printing

**Figure 22:**
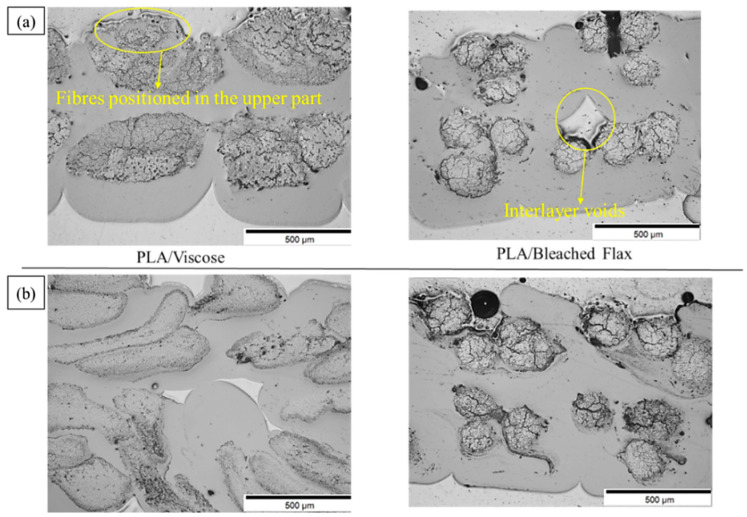
Optical microscope images of 3D-printed composites produced from (**a**) solution impregnation and (**b**) emulsion impregnation.

Figure 22a,b presents optical microscopy images of the cross-sections of solution- and emulsion-impregnated 3D-printed composites, respectively. For all composites, except the emulsion-impregnated PLA/viscose, the reinforcing yarns were positioned toward the upper part of the printing layer. This positioning may be due to the nozzle’s drag force, which can pull the fibres during printing. Similar observations have been reported in multiple studies on the 3D printing of long/continuous fibre-reinforced composites [4,6,9,51]. The absence of surface-exposed fibres in the emulsion-impregnated PLA/viscose composites suggests enhanced matrix impregnation and interfacial bonding, preventing the yarn from being pulled from the matrix [36]. Table 4 summarises the porosity and fibre weight percentages of the 3D-printed composites, which match the fibre content percentages of the filaments. A slight increase in porosity was observed for all printed specimens compared to the 3D printing filaments, likely due to interlayer defects introduced during the printing process, as shown in Figure 22. These voids, often created by increased layer heights, are known to negatively impact the tensile properties of 3D-printed composites [4,52].

Table 5 provides a summary of the tensile properties of the 3D-printed PLA/viscose composites along the fibre direction. All printed samples exhibited notable reductions in tensile properties compared to the filaments, likely due to interlayer porosities introduced during printing and a possible decrease in crystallinity. The use of higher layer heights on a heated build platform may reduce molecular orientation during deposition, resulting in the formation of less stable δ-form crystallites of PLA [53]. The emulsion-impregnated PLA/viscose composite achieved the highest tensile strength, which aligns with its improved fibre distribution and lower porosity levels.

This study marks the first to successfully 3D print PLA/continuous viscose composites, whereas PLA/flax yarn composites have been previously explored. Compared to the existing literature on PLA/flax yarn composites, the present results indicate lower tensile properties for PLA/viscose [35,54]. Modifications to 3D printing equipment, such as printing with lower layer heights and smaller filament diameters, could help minimise tensile property losses [55,56]. Additionally, this research involved adapting filaments for use with a commercial 3D printer, highlighting practical applicability and paving the way for high-strength filaments suitable for large-scale 3D printing. A key advantage of the developed filaments is their potential for use with larger nozzle sizes, significantly enhancing productivity and enabling the printing of larger objects [9].

**Figure 23:**
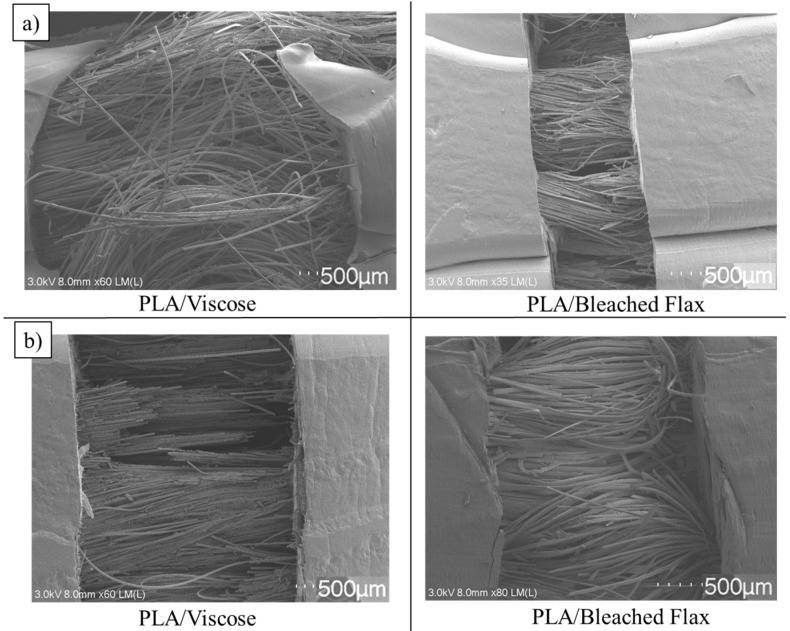
SEM images of fracture surfaces of tensile-tested 3D-printed specimens: (**a**) solution impregnation; (**b**) emulsion impregnation (scale bar = 500 µm).

The fracture behaviour of the tensile-tested composite specimens was similar to that observed in the 3D printing filaments, displaying numerous fibre pullouts and a brittle fracture of the surrounding matrix, as shown in Figure 23. Emulsion-impregnated PLA/viscose composites demonstrated fewer fibre pullouts and more fibre breakage compared to other composites, which correlates with their superior tensile properties [57,58].

**Figure 24:**
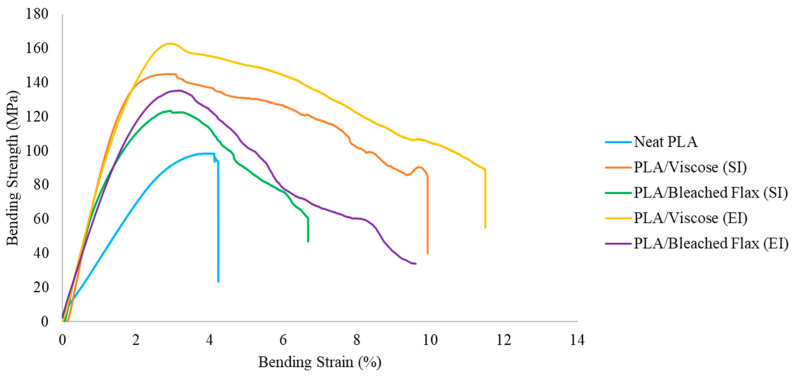
Fourteen stress vs. strain for flexural testing of 3D-printed composites; SI—solution impregnation; EI—emulsion impregnation.

Table 6 summarises the flexural properties of FDM 3D-printed composites, with the corresponding stress–strain curves shown in Figure 24. Neat PLA exhibited a linear stress–strain response, followed by a sudden fracture due to its brittle characteristics [59]. In contrast, the composite specimens demonstrated a linear stress–strain response, followed by a gradual decline in strength with increasing strain, indicative of fibre/matrix interface debonding prior to failure [60,61,62]. Emulsion impregnation resulted in greater improvements in the flexural properties of PLA/viscose composites, while PLA/bleached flax composites showed better performance with solution impregnation, consistent with the trends observed in tensile testing. The most significant enhancement in flexural properties compared to neat PLA was seen with emulsion-impregnated PLA/viscose, which achieved a 79.1% increase in bending strength and a 227% increase in bending modulus.

Solution-impregnated PLA/viscose composites demonstrated exceptional impact strength, showing a 502% increase compared to neat PLA. Similarly, solution- and emulsion-impregnated PLA/bleached flax composites displayed comparable impact strengths, with increases of 214% and 221.5%, respectively, over neat PLA. Notably, the impact strength of emulsion-impregnated PLA/viscose samples exceeded the upper limit of the Charpy impact testing machine and thus is reported as >127 kJ/m^2^ in Table 7. Based on the findings of this study, the Charpy impact strengths obtained here surpass any reported values for bio-derived fibre-reinforced PLA composites to date [63,64,65,66,67,68,69].

The energy absorption mechanisms help explain the higher impact strength observed in PLA/viscose composites. It is hypothesised that, rather than breaking, the fibres tend to pull out, a process that requires significant energy. Additional energy is dissipated through debonding at the fibre/matrix interface and frictional sliding between the fibres and the matrix. Weak interfaces can also deflect cracks, extending the crack path and thereby increasing the energy needed for crack propagation. The matrix may also plastically deform or experience microcracking, which further absorbs energy [70]. As a result, fibre pullout, debonding, crack deflection, frictional sliding, and matrix deformation all contribute to multiple energy dissipation mechanisms, which together enhance the composite’s impact strength [67,71].

## 4. Conclusions

In this study, we developed effective processes for solution and emulsion impregnation and consolidation, leading to the production of high-quality composite filaments. Emulsion-impregnated composites demonstrated greater polymer uptake, attributed to the enhanced affinity between water and bio-derived fibres. In contrast, solution-impregnated composites showed superior polymer distribution, especially in twisted flax-reinforced filaments, due to efficient impregnation with the PLA/DCM solution. The emulsion-impregnated PLA/viscose composites achieved n tensile strength (254.7 MPa) and Young’s modulus (9.1 GPa), surpassing values reported in the literature. Similarly, the solution-impregnated PLA/bleached flax composites attained the highest tensile strength (356.1 MPa), exceeding existing values for PLA/flax composites. However, when producing 3D printing filaments by combining multiple filaments and using melt impregnation, a reduction in tensile properties occurred due to the additional polymer from melt impregnation required for printability. Increased porosity from the 3D printing process further contributed to this reduction. Despite this, the emulsion-impregnated PLA/viscose composites displayed the highest tensile and flexural properties, underscoring the importance of efficient impregnation methods. The combination of strong tensile properties and high impact strength (greater than 127 kJ/m² for viscose-based composites) broadens the applicability of PLA-based composites and opens new possibilities for large-format 3D printing of structural components.

## Figures and Tables

**Table 1:** Tensile properties of single fibres.

Fibre	Viscose			Bleached Flax		
Property	Tensile Strength (MPa)	Young’s Modulus (GPa)	Strain at Break (%)	Tensile Strength (MPa)	Young’s Modulus (GPa)	Strain at Break (%)
Avg.	724.2	22.8	13.2	921.6	30.7	3.3
Std Dev	187.8	5.97	4.2	320.2	12.2	1.6

**Table 2:** Solution and emulsion impregnation formulations.

**Formulation**	**Solution Impregnation**
10 wt% x1	Single impregnation cycle with 10 wt% PLA/DCM solution
10 wt% x2	Two single impregnation cycles with 10 wt% PLA/DCM solution
7 wt% x2	Two single impregnation cycles with 7 wt% PLA/DCM solution
7 wt% x3	Three single impregnation cycles with 7 wt% PLA/DCM solution
7 wt% x1 (tandem)	One tandem impregnation cycle with 7 wt% PLA/DCM solution
7 wt% x2 (tandem)	Two tandem impregnation cycles with 7 wt% PLA/DCM solution
**Formulation**	**Emulsion Impregnation**
Emulsion x1	Single impregnation cycle with 40 wt% PLA/water emulsion
Emulsion x2	Two single impregnation cycles with 40 wt% PLA/water emulsion
Emulsion x3	Three single impregnation cycles with 40 wt% PLA/water emulsion
Emulsion x1 (tandem)	One tandem impregnation cycle with 40 wt% PLA/water emulsion
Emulsion x2 (tandem)	Two tandem impregnation cycles with 40 wt% PLA/water emulsion

**Table 3:** Summary of the tensile properties of single-yarn composite formulations used for the production of 3D printing filaments (n = 5, values are presented as mean ± SD).

Reinforcement	Processing Condition	Fibre wt%	Tensile Strength (MPa)	Young’s Modulus (GPa)	Strain at Break (%)
Viscose	7 wt% x2	65.5 ± 2.0	240.8 ± 24.6	7.9 ± 1.5	15.4 ± 2.5
	Emulsion x2	41.2 ± 1.3	254.7 ± 15.3	9.1 ± 0.4	14.7 ± 2.1
Bleached Flax	7 wt% x2 (tandem)	60.0 ± 0.2	326.1 ± 15.5	17.6 ± 0.8	3.2 ± 0.3
	Emulsion x2 (tandem)	41.0 ± 0.5	296.2 ± 22.1	12.6 ± 1.1	3.2 ± 0.2

**Table 4:** Fibre and polymer weight percentage and porosity percentage of 3D-printed composite specimens (n = 5, values are presented as mean ± SD).

Impregnation Type	Composite Filament	Fibre wt%	Porosity (%)
Solution Impregnation	PLA/Viscose	33.4 ± 1.3	6.3 ± 3.4
	PLA/Bleached Flax	27.8 ± 2.1	8.8 ± 1.2
Emulsion Impregnation	PLA/Viscose	32.8 ± 0.9	7.0 ± 2.4
	PLA/Bleached Flax	26.4 ± 2.8	9.2 ± 0.5

**Table 5:** Tensile properties of 3D-printed composite specimens and percentage decrease compared to 3D printing filaments (highlighted in parentheses) (n = 5, values are presented as mean ± SD).

Impregnation Type	Material	Fibre wt%	Tensile Strength (MPa) (% Decrease)	Young’s Modulus (GPa) (% Decrease)	Strain at Break (%) (% Decrease)
Solution Impregnation	PLA/Viscose	33.4 ± 1.3	139.9 ± 10.8 (30.7%)	5.9 ± 2.3 (13.2%)	7.5 ± 2.3 (57.1%)
	PLA/Bleached flax	27.8 ± 2.1	127.3 ± 8.8 (29.6%)	7.2 ± 0.5 (25.0%)	3.9 ± 0.7 (23.0%)
Emulsion Impregnation	PLA/Viscose	32.8 ± 0.9	158.0 ± 10.5 (17.0%)	6.9 ± 0.7 (6.8%)	8.3 ± 0.5 (62.3%)
	PLA/Bleached flax	26.4 ± 2.8	108.4 ± 12.1 (29.9%)	5.8 ± 1.0 (36.5%)	3.2 ± 0.3 (5.9%)

**Table 6:** Flexural properties of FDM 3D-printed composites (n = 5, values are presented as mean ± SD).

Impregnation Type	Material	Bending Strength (MPa)	Bending Modulus (GPa)
None	Neat PLA	91.0 ± 5.5	3.0 ± 0.2
Solution Impregnation	PLA/Viscose	154.3 ± 20.3	7.9 ± 2.1
	PLA/Bleached flax	127.0 ± 20.3	8.9 ± 1.0
Emulsion Impregnation	PLA/Viscose	163.8 ± 20	9.8 ± 1.1
	PLA/Bleached flax	122.4 ± 17.5	8.5 ± 1.4

**Table 7:** Impact strength of FDM 3D-printed composites (n = 5, values are presented as mean ± SD).

Impregnation Type	Material	Impact Strength (kJ/m^2^)
None	Neat PLA	20.9 ± 4.5
Solution Impregnation	PLA/Viscose	125.8 ± 2.1
	PLA/Bleached flax	65.6 ± 5.1
Emulsion Impregnation	PLA/Viscose	>127
	PLA/Bleached flax	67.2 ± 5.0

## Data Availability

The original contributions presented in the study are included in the article/Supplementary Materials, further inquiries can be directed to the corresponding author.

## Supplementary Materials

The following supporting information can be downloaded at: https://www.mdpi.com/article/10.3390/ma17225554/s1, Figure S1: Schematic of single fibre tensile testing; Figure S2: System compliance of the tensile testing; Figure S3: (a) Optical microscopy of PL 1005 emulsion (b) Production of PL 1005 filament; Figure S4: TGA and DSC of PLA2003D and PL1005; Figure S5: SEM images of reinforcement yarns and single fibres (a) Viscose (b) Bleached flax; Figure S6: Stress vs strain curves of viscose and bleached flax single fibres; Figure S7: Box plot of tensile properties of viscose single fibres (a) Tensile strength (b) Young’s modulus (c) Elongation at break; Figure S8: Box plot of tensile properties of bleached flax single fibres (a) Tensile strength (b) Young’s modulus (c) Elongation at break; Figure S9: Thermogravimetric analysis (TGA) and corresponding DTG of bleached flax and viscose fibres; Figure S10: SEM images of cryofracture surfaces of solution impregnated and consolidated PLA/viscose filaments.; Figure S11: SEM images of cryofracture surfaces of solution impregnated and consolidated PLA/bleached flax filaments; Figure S12: SEM images of cryofracture surfaces of emulsion impregnated and consolidated PLA/viscose filaments; Figure S13: SEM images of cryofracture surfaces of emulsion impregnated and consolidated PLA/bleached flax filaments; Figure S14: Optical microscope images of consolidated filaments (solution impregnation) for different formulations—PLA/viscose; Figure S15: Optical microscope images of consolidated filaments (solution impregnation) for different formulations—PLA/bleached flax; Figure S16: Optical microscopy images of consolidated filaments for different emulsion impregnation formulations—PLA/viscose; Figure S17: Optical microscopy images of consolidated filaments for different emulsion impregnation formulations—PLA/bleached flax; Figure S18: DSC thermograms of 3D printing composite filaments (a) Solution impregnation (b) Emulsion impregnation; Figure S19: DSC thermograms of FDM 3D printed composites (a) Solution impregnation (b) Emulsion impregnation; Table S1: Properties of PLA 2003D and PL1005 (supplier-provided values are highlighted in parentheses; NA—not available); Table S2: Tensile properties of single fibres; Table S3: Fibre and polymer weight percentage and porosity percentage of composite filaments produced using solution impregnation and consolidation method; Table S4: Fibre and polymer weight percentage and porosity percentage of composite filaments produced using emulsion impregnation and consolidation method; Table S5: Tensile properties of composite filaments produced from solution impregnation and consolidation method; Table S6: Tensile properties of composite filaments produced using emulsion impregnation and consolidation method. Table S7: Fibre and polymer weight percentage and porosity percentage of multiple consolidated filaments; Table S8: Fibre and polymer weight percentage and porosity percentage of melt impregnated 3D printing filaments; Table S9: Tensile properties of multiple consolidated filaments; Table S10: Tensile properties of 3D printing filaments; Table S11: Summary of DSC for 3D printing composite filaments; Table S12: Summary of DSC of 3D printed composites; References [72,73,74,75,76] are cited in the supplementary materials.
