# Supplementary material for: Polylactide Composites Reinforced with Pre-Impregnated Natural Fibre and Continuous Cellulose Yarns for 3D Printing Applications
Source: Materials (Basel). 2024 Nov 14;17(22):5554. doi: 10.3390/ma17225554 (PMC11595717; doi:10.3390/ma17225554)
Supplement: Supplementary file 1 [file materials-17-05554-s001.zip › materials-3311804-supplementary.pdf]

Supplementary information

1 – Materials and methods (not shown in the main manuscript)

1.1 - Single fibre tensile testing

Viscose is a continuous bio-derived fibre, whereas flax is a plant fibre. The main difference between these fibres is that viscose is a solid fibre throughout its cross-section, and flax fibre comprises a solid region and the lumen, a hollow structure in the middle [1]. Flax fibres are assumed to be solid throughout the cross-section, and both flax and viscose fibres are assumed to have circular cross-sections for this experiment. These assumptions enable comparison with most existing literature for mechanical testing of single fibres [2,3].

Tensile properties of viscose, standard flax, and bleached flax fibres were obtained using the single fibre tensile test method according to ASTM D3379 standard [4]. Single fibres were separated from yarns and mounted on 350 GSM paper tabs using gauge lengths of 2mm, 5mm, 7mm, and 10mm, respectively. Fifteen samples were tested for each gauge length. Epoxy was used to mount the fibres to the paper tabs, and the samples were subsequently cured for 4 hours at room temperature, followed by conditioning in a climatic chamber at 23°C and relative humidity of 50% for 48 hours. The diameters of the fibres were measured using an Olympus BX60F5 (Tokyo, Japan) optical microscope. The diameter of each fibre was measured at five different points, and the average value was used for testing and calculations. Instron® 5982 UTM (universal testing machine) (Norwood, MA, USA) was used to perform the tensile test after cutting the supporting sides of the tabs, as shown in Figure S1. A crosshead displacement rate of 2mm/min and a load cell of 10N was used.

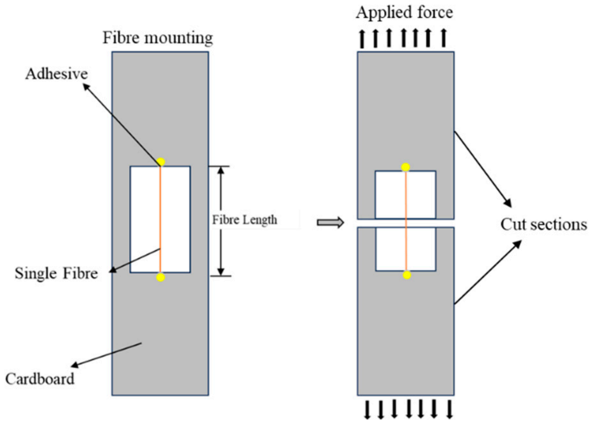

Figure S1 - Schematic of single fibre tensile testing

The determination of Young's modulus of a single fibre from tensile test data requires correction of displacement using the compliance correction method. The load cell of the tensile tester measures the load applied onto the specimen; most commonly, an extensometer or strain gauge is used to measure displacement. However, these tools cannot be applied to single fibres because their diameters are too small. Instead, displacement is read directly from the crosshead movement of the tensile testing machine, which includes fibre elongation and the deformations in the crosshead. The actual displacement of the fibres due to the compliance of the testing system can be calculated by using a correction factor.

The system compliance or the correction factor (Cs) was found by choosing the viscose fibre as the material with known Young's modulus (19 GPa). The apparent compliance Ca was calculated for each length of single fibres tested by inverting the gradient obtained from the force vs displacement curves. The value obtained for C_a was extrapolated to gauge the length of zero, as shown in Figure S2, to obtain system compliance C_s.

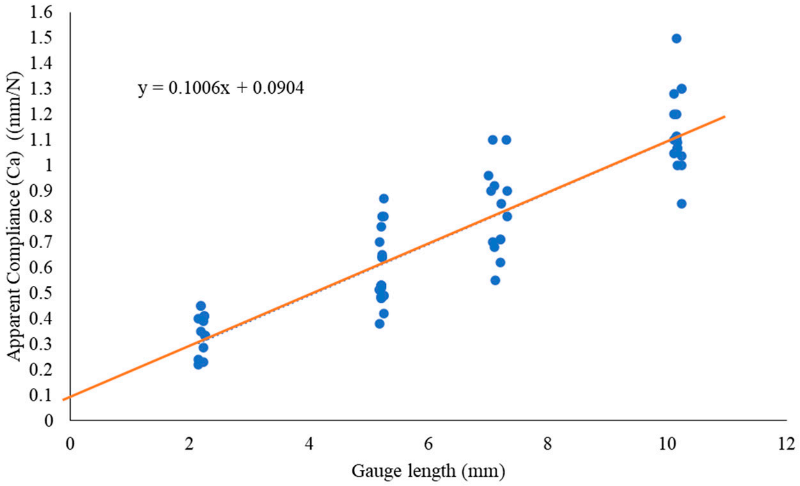

Figure S2 - System compliance of the tensile testing

The true compliance C was then calculated as

$$C = C_a - C_s \tag{S1}$$

The Young's modulus was calculated using the equation,

$$E_f = \frac{L}{CA} \tag{S2}$$

where L is the specimen gauge length, C is the true compliance, and A is the average fibre cross-sectional area.

1.2 – Matrix tensile testing

Filaments were produced from PLA 2003D and PL1005 grades for tensile testing. PLA 2003D is available in the form of granules which were fed to the Filabot EX2 (Barre, Vermont, USA) single screw

extruder, and a filament of diameter 1.75 ± 0.5 mm was extruded at a temperature of 180°C. PL 1005 is a commercial water-based emulsion which consists of PLA particles (diameter of 5μm) dispersed in water, as shown in Figure S3 (a). The emulsion was air-dried in the fume hood for 48 hours. The PLA powder obtained (Figure S3 (b)) was then dried in the vacuum oven for 24 hours at 30°C and 3 hours at 50°C. A moisture analysis was done using KERN and Sohn GmbH moisture analyser (Balingen, Germany) to ensure the moisture was removed entirely from the PLA powder. The analysis protocol involved measuring the weight loss in the powder at a temperature of 102°C with an accuracy of 0.01%. The Filabot single-screw extruder was then used to produce the filament with an extrusion temperature of 190°C and a diameter of 1.75 ± 1 mm. The filaments were further granulated to produce pellets of 4mm length using a Moretto GR knife mill (Mercer County, PA, USA). The pellets were used to perform a second extrusion to improve the filament diameter tolerance, as shown in Figure S3. Filaments produced from the second extrusion had a diameter with a tolerance of 1.75 ± 0.5 mm. Tensile testing of the filaments was done using an Instron® 5982 tensile tester (Massachusetts, USA) with a 5 kN load cell using a 10 mm extensometer at a crosshead displacement of 5 mm/min. A gauge length of 30 mm was used for filament testing. Six repeats for each material were tested, and the samples were conditioned in a climatic chamber at 23°C and relative humidity of 50% for 48 hours before testing.

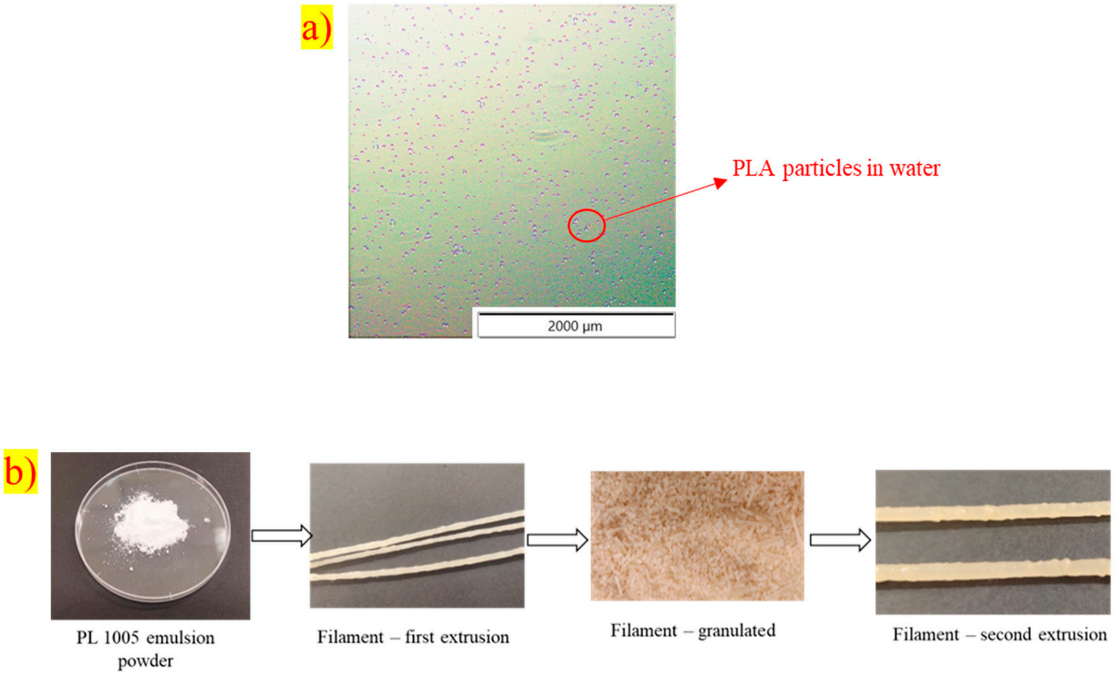

Figure S3 - (a)Optical microscopy of PL 1005 emulsion (b) Production of PL 1005 filament

1.3 - Differential scanning calorimetry (DSC)

The calorimetric measurements of PLA and composites were recorded using a Netzsch DSC3500 Sirius differential scanning calorimeter (Selb, DE) using aluminium crucibles from 20-200 °C at 10 °C/min with a Nitrogen flow of 60 mL/min. The glass transition (T_g), melting (T_m), and cold crystallisation (T_{cc}) temperatures were obtained from the DSC scans. The PLA crystallinity of the samples was determined according to Equation S3 [5]:

$$X_c = \frac{(\Delta H_m - \Delta H_{cc})}{\Delta H_f \times X_{PLA}} . 100 \qquad \text{(S3)}$$

where ΔH_m and ΔH_c are the enthalpies of melting and cold crystallisation, respectively, ΔH_f is the melting enthalpy of 100% crystalline PLA (93 J/g) [5], and X_{PLA} is the mass fraction of PLA in the composite.

1.4 – Thermogravimetric analysis (TGA)

Thermal properties of the reinforcements and matrix were analysed using a Perkin Elmer STA8000 (Connecticut, USA) thermogravimetric analyser from 30 °C to 600 °C at a heating rate of 10 °C/min under an argon flow of 40 mL/min.

2 – Additional information on the PLA matrices

Table S1 - Properties of PLA 2003D and PL1005 (supplier-provided values are highlighted in parentheses; NA – not available)

Material	Tensile strength (MPa)	Young's modulus (GPa)	Elongation at break (%)
**PLA 2003D**	59.8±3.1 (60)	3.7±0.8 (3.5)	3.9±0.6 (6)
**PL1005**	49.2±4.4 (10)	2.9±0.2 (NA)	3.5±0.9 (NA)

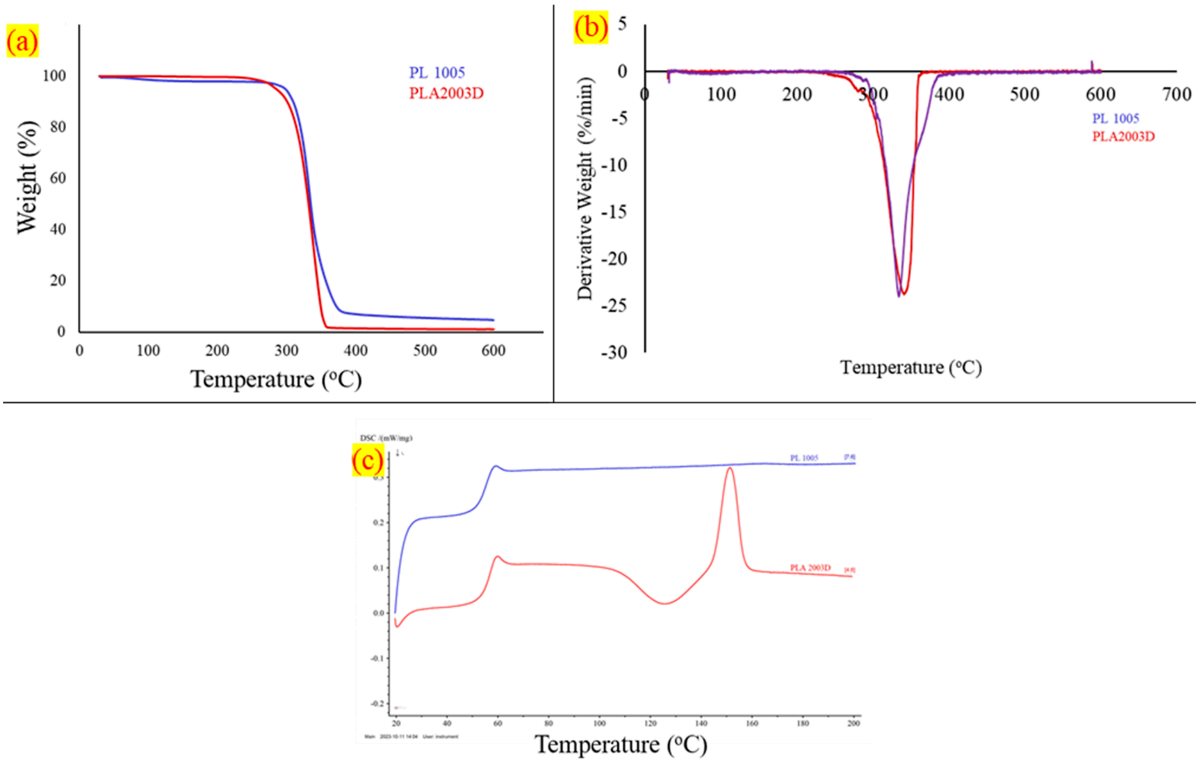

Figure S4 – TGA and DSC of PLA2003D and PL1005

3 - Characterisation of reinforcing fibres

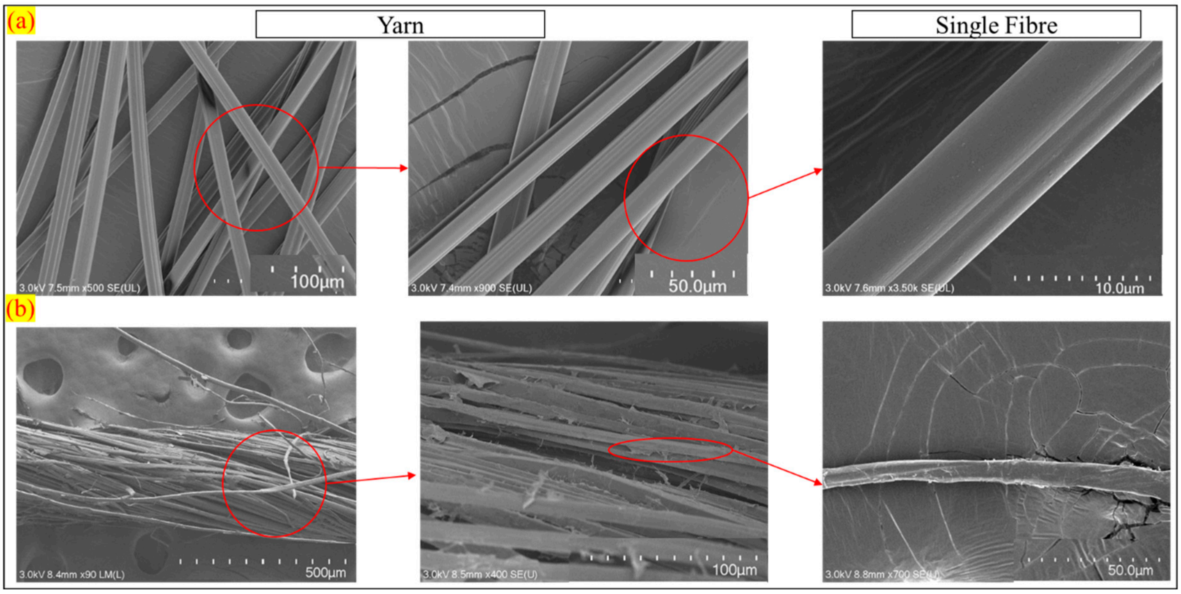

Figure S5 - SEM images of reinforcement yarns and single fibres a) Viscose b) Bleached flax

Table S2 - Tensile properties of single fibres

Fibre	Viscose			Bleached Flax		
Property	TS (MPa)	YM (GPa)	ε_b (%)	TS (MPa)	YM (GPa)	ε_b (%)
Mean	724.2	22.8	13.2	921.6	30.7	3.3
Std Dev	187.8	5.97	4.2	320.2	12.2	1.6

TS=Tensile Strength; YM=Young's modulus; ε_b = Strain at break

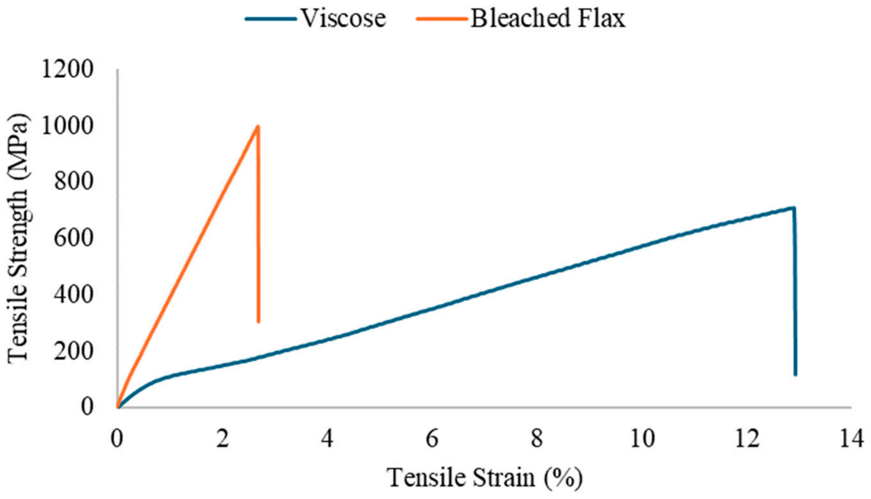

Figure S6 - Stress vs strain curves of viscose and bleached flax single fibres.

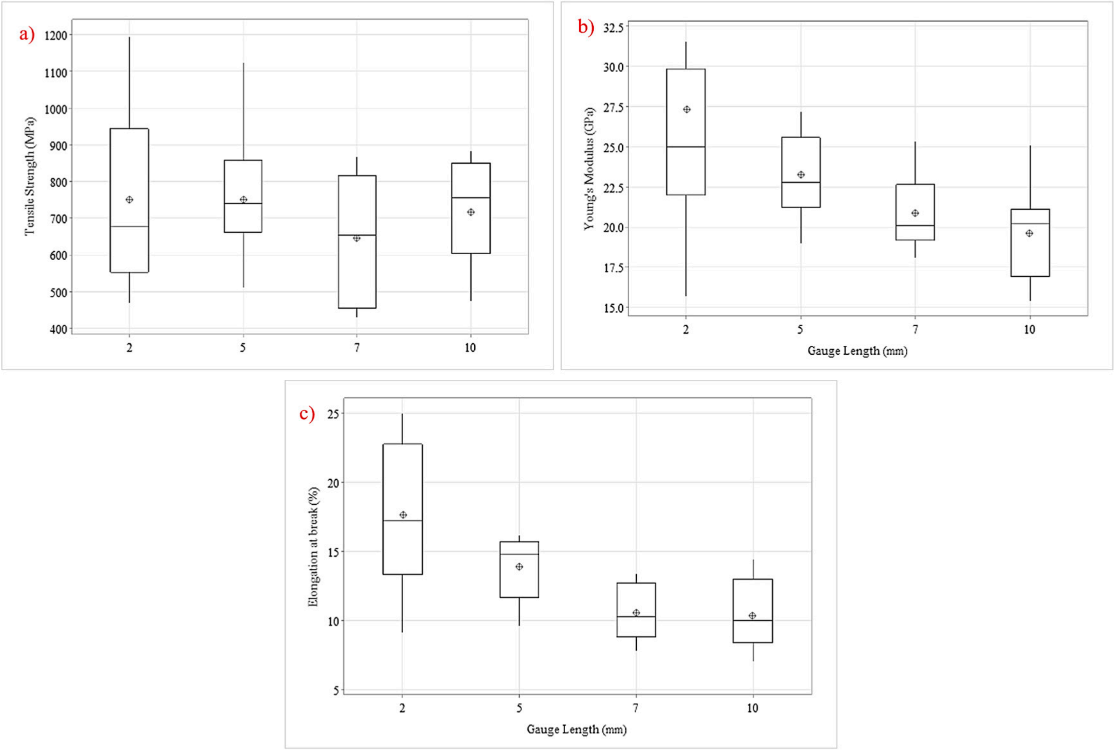

Figure S7 - Box plot of tensile properties of viscose single fibres a) Tensile strength b) Young's modulus c) Elongation at break

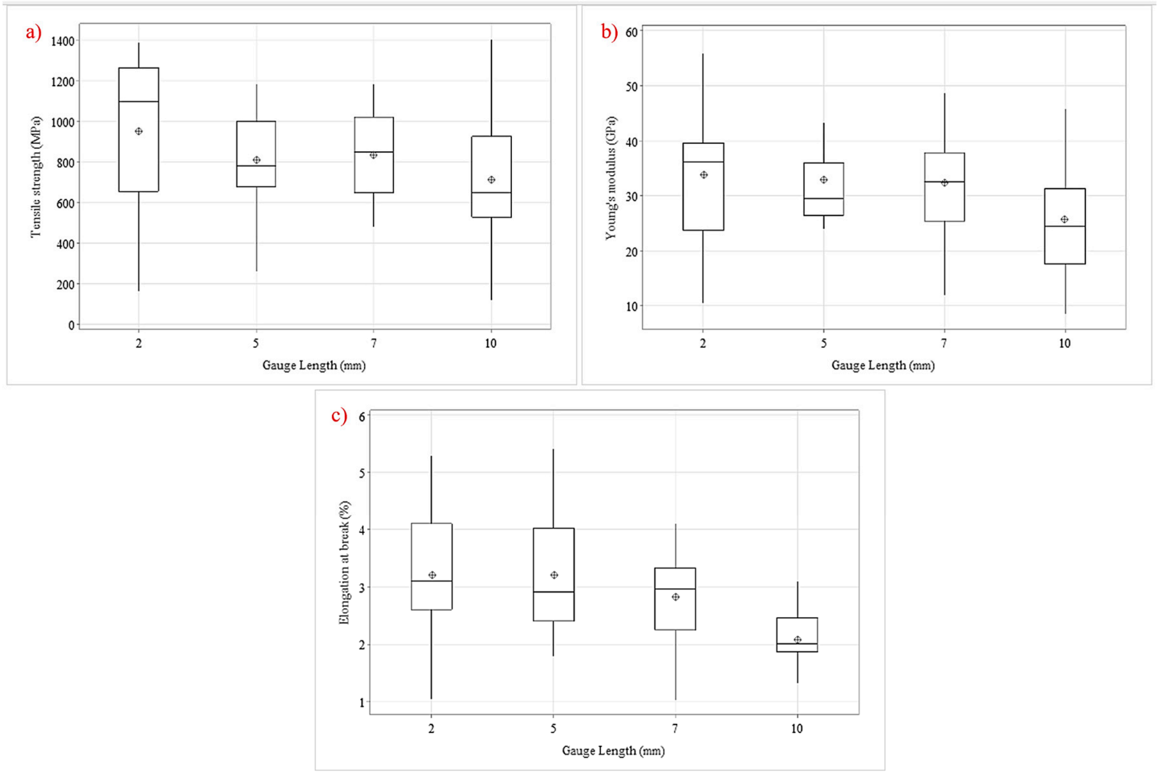

Figure S8 - Box plot of tensile properties of bleached flax single fibres a) Tensile strength b) Young's modulus c) Elongation at break

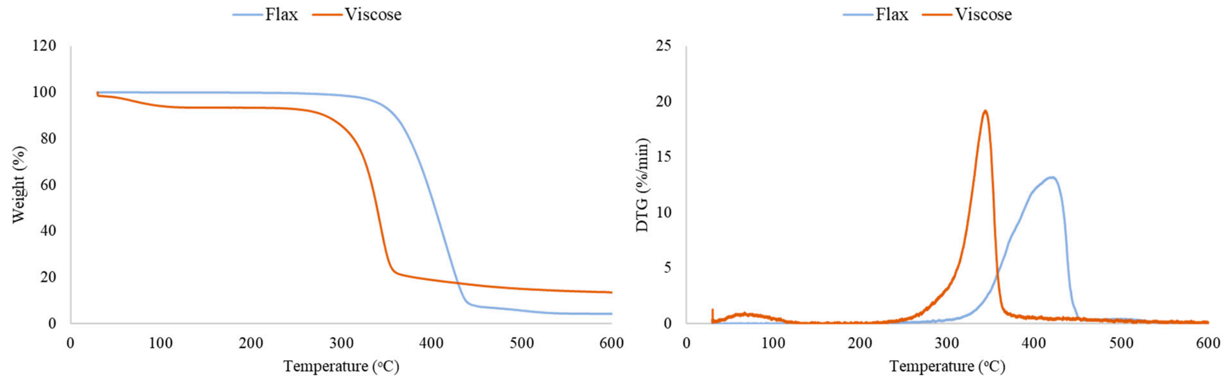

Figure S9 – Thermogravimetric analysis (TGA) and corresponding DTG of bleached flax and viscose fibres.

4 – SEM analysis of cryofracture single yarn impregnated filaments

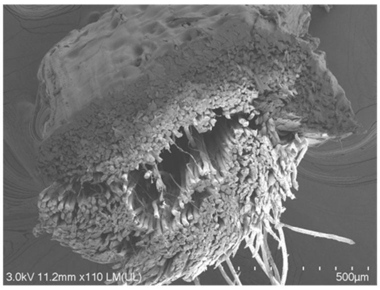 10%PLA/DCM x1/ Viscose	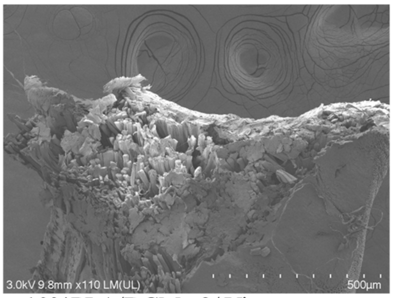 10%PLA/DCM x2/ Viscose	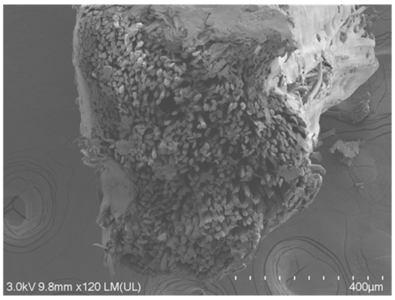 7%PLA/DCM x2/ Viscose (single)
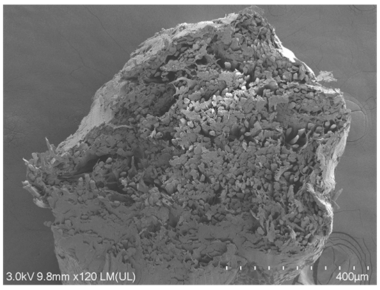 7%PLA/DCM x3/ Viscose (single)	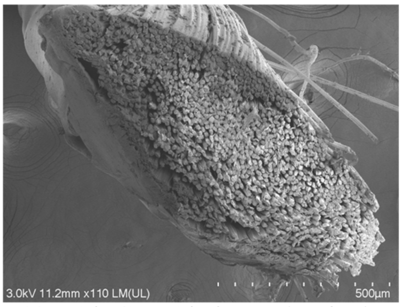 7%PLA/DCM x2/ Viscose (tandem)	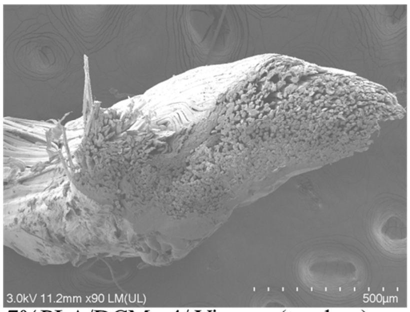 7%PLA/DCM x4/ Viscose (tandem)

Figure S10 – SEM images of cryofracture surfaces of solution impregnated and consolidated PLA/viscose filaments.

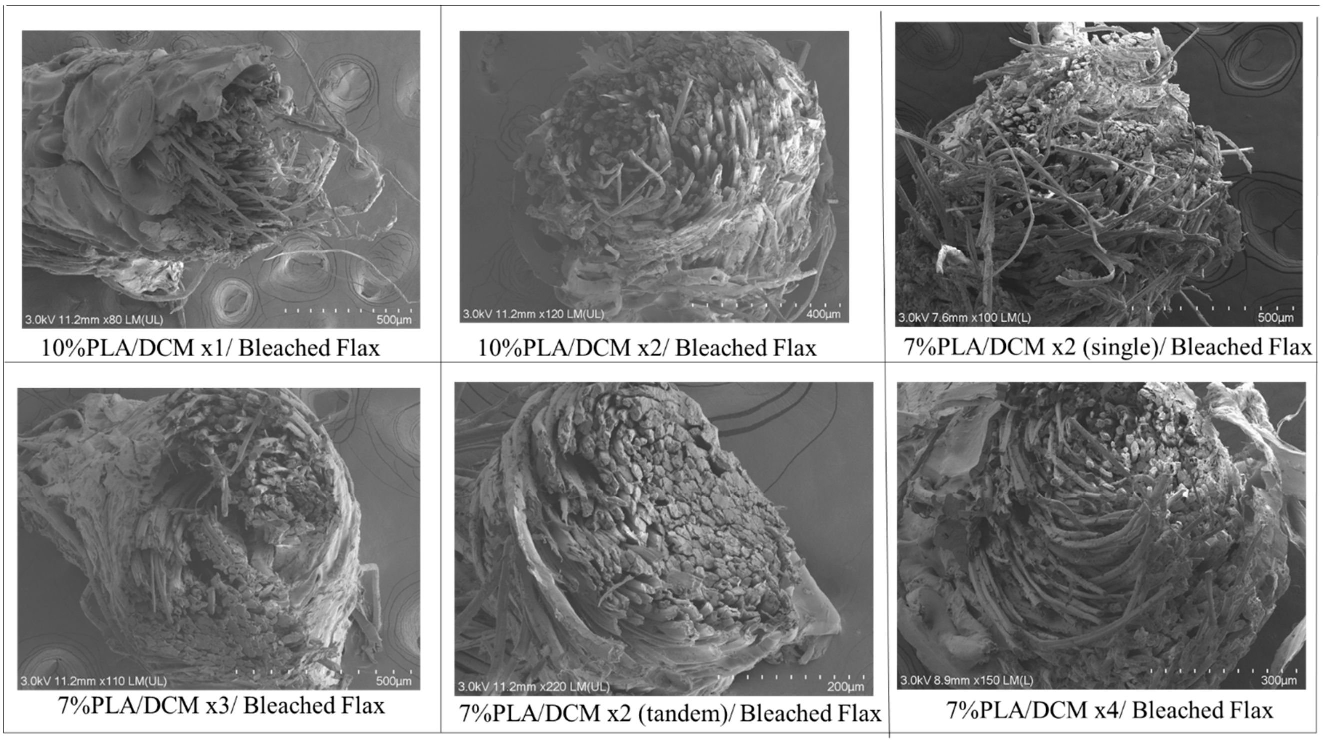

Figure S11 - SEM images of cryofracture surfaces of solution impregnated and consolidated PLA/bleached flax filaments.

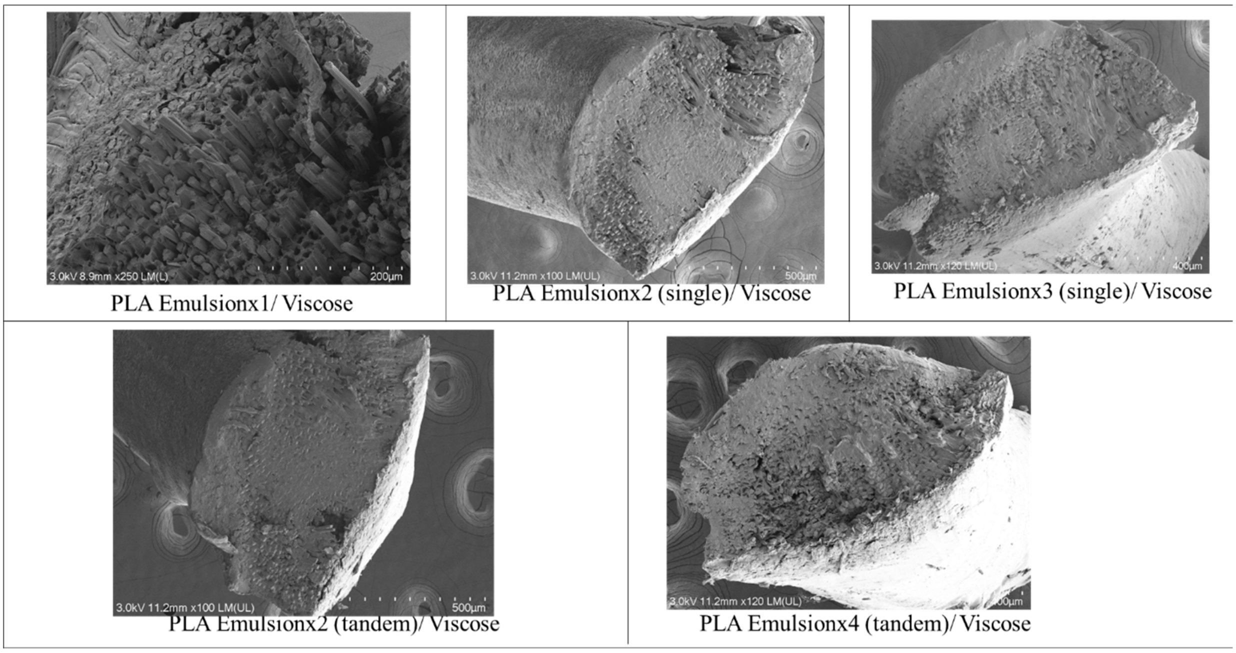

Figure S12 - SEM images of cryofracture surfaces of emulsion impregnated and consolidated PLA/viscose filaments.

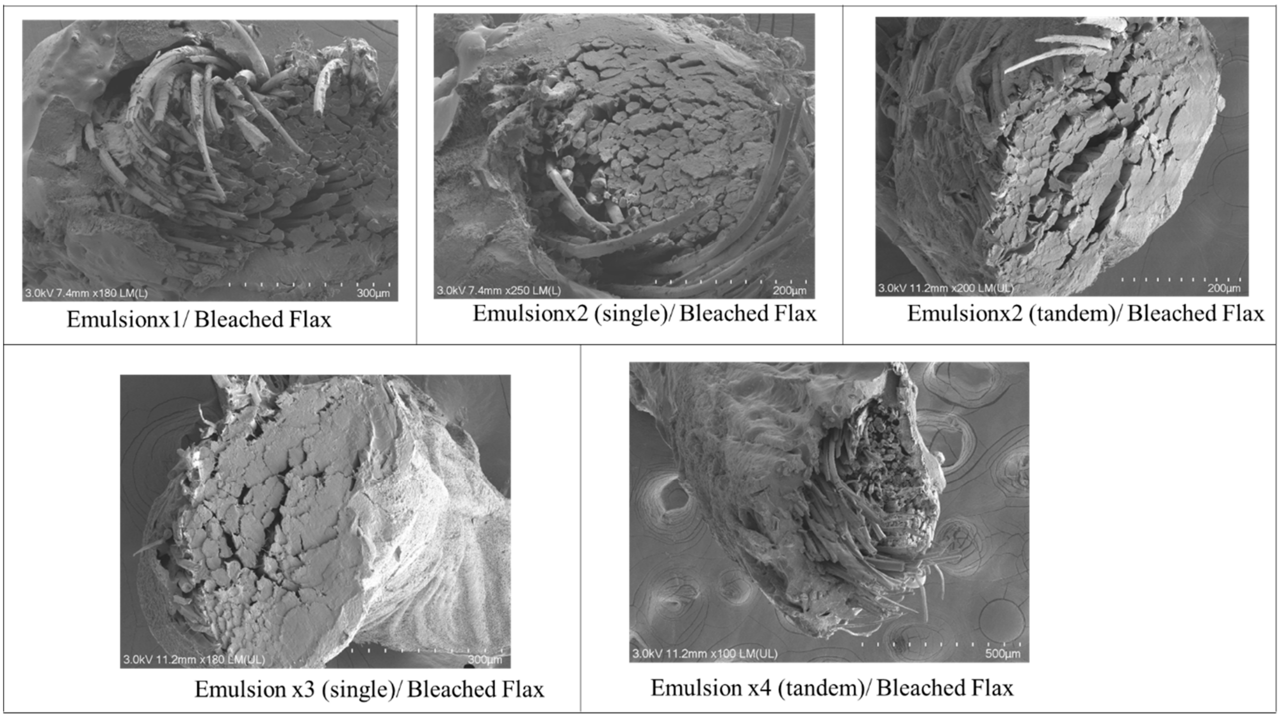

Figure S13 - SEM images of cryofracture surfaces of emulsion impregnated and consolidated PLA/bleached flax filaments.

4 – Optical microscopy analysis of single yarn impregnated filaments

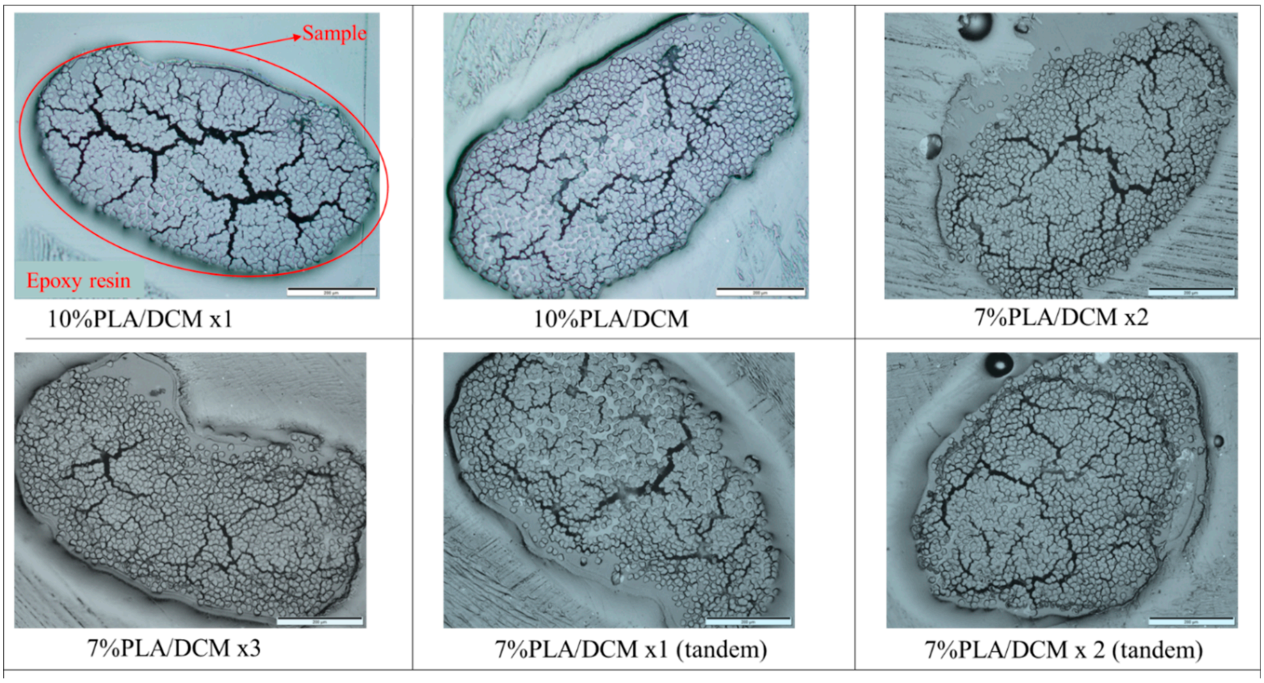

Figure S14 - Optical microscope images of consolidated filaments (solution impregnation) for different formulations – PLA/viscose

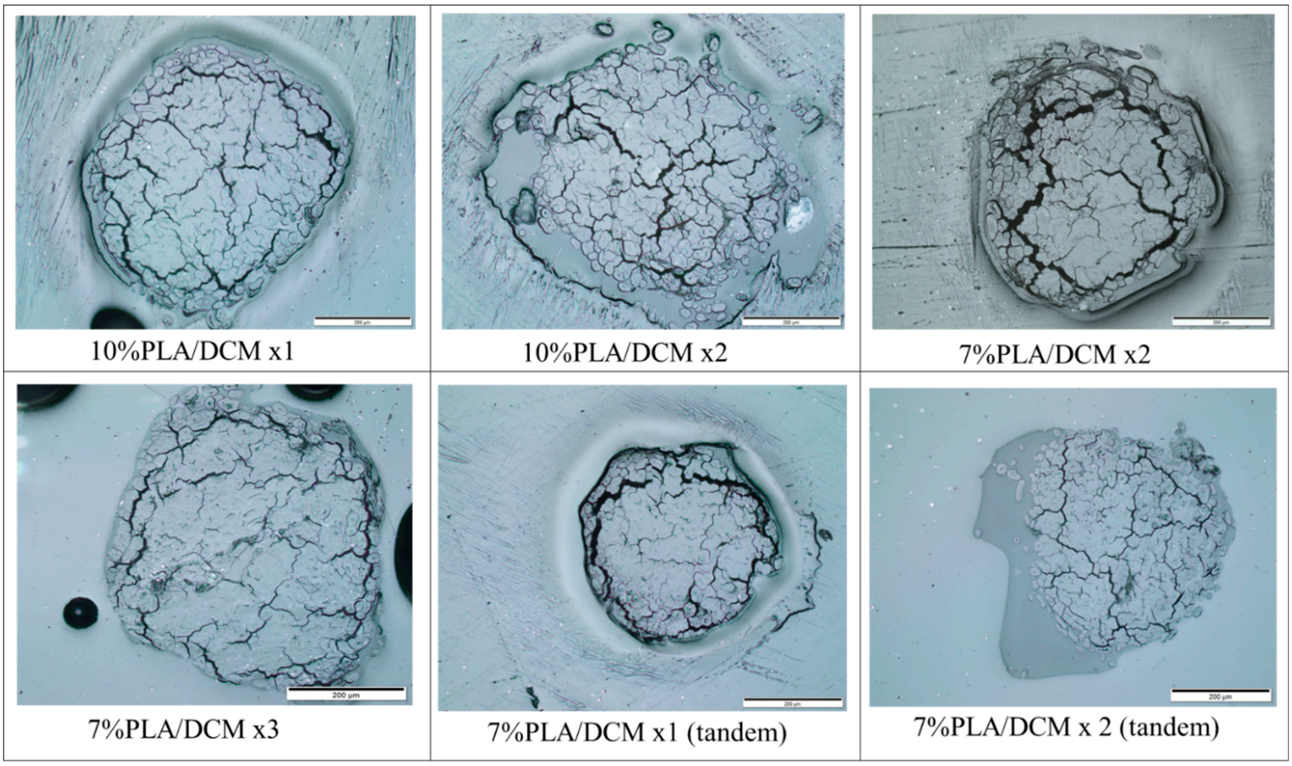

Figure S15 - Optical microscope images of consolidated filaments (solution impregnation) for different formulations - PLA/bleached flax

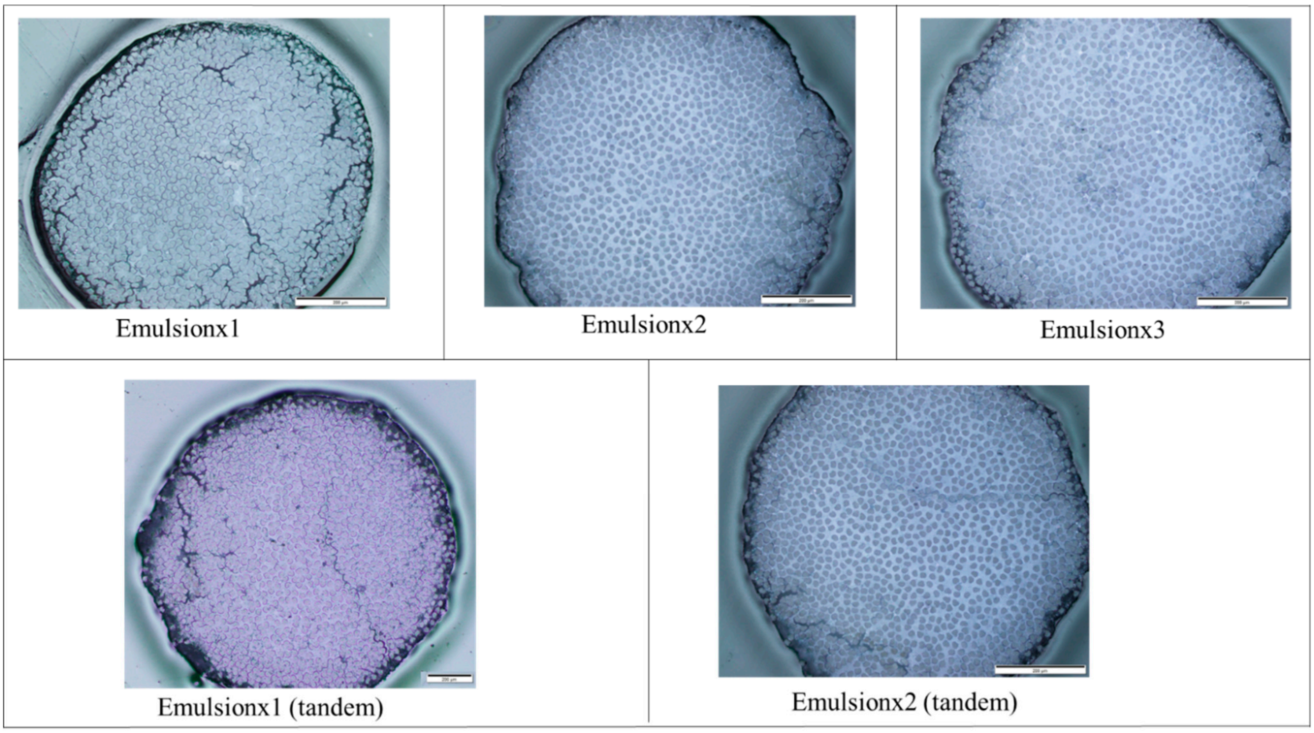

Figure S16 - Optical microscopy images of consolidated filaments for different emulsion impregnation formulations – PLA/viscose

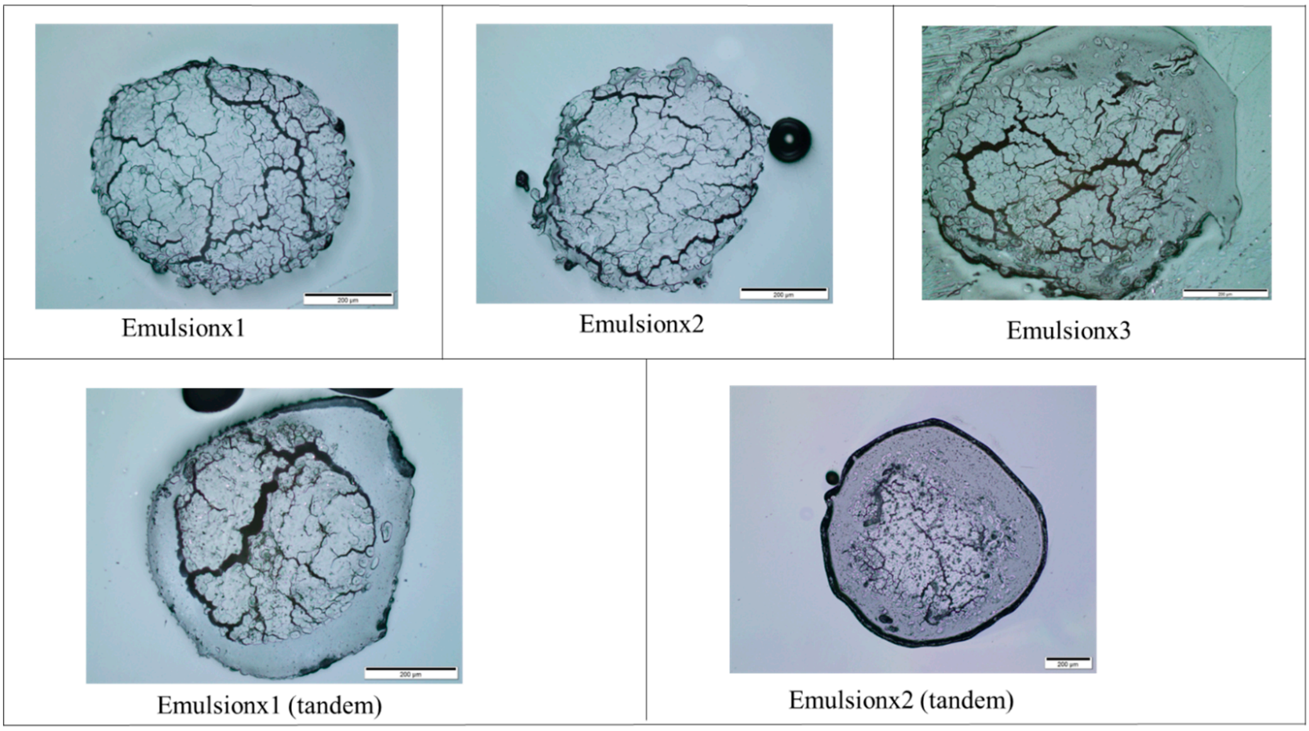

Figure S17 - Optical microscopy images of consolidated filaments for different emulsion impregnation formulations – PLA/bleached flax

5 – Porosity and reinforcement wt% of single yarn reinforced composites

Table S3 - Fibre and polymer weight percentage and porosity percentage of composite filaments produced using solution impregnation and consolidation method.

Formulation	Composite Filament	Wt % of Reinforcement	Wt % of Polymer	Porosity (%)
**10wt%x1**	PLA/Viscose	76.8±0.2	23.2 ± 0.2	19.8±1.8
	PLA/Bleached Flax	77.8±0.2	22.2 ± 0.2	14.5±4.6
**10wt%x2**	PLA/Viscose	63.2±0.9	36.8 ± 0.9	20.2±2.4
	PLA/Bleached Flax	72.2±1.2	27.8 ± 1.2	14.9±4.2
**7wt%x2**	PLA/Viscose	65.5±2.0	34.5 ± 2.0	14.2±4.5
	PLA/Bleached Flax	74.80±1.4	25.20 ± 1.4	12.8±9.3
**7wt%x3**	PLA/Viscose	68.1±0.6	31.9 ± 0.6	9.5±3.6
	PLA/Bleached Flax	64.3±0.1	35.7 ± 0.1	6.5±3.7
**7wt%x1 (tandem)**	PLA/Viscose	70.8±0.6	29.2 ± 0.6	14.7±2.6
	PLA/Bleached Flax	69.2±0.2	30.8 ± 0.2	13.3±8.4
**7wt%x2 (tandem)**	PLA/Viscose	64.3±1.0	35.7 ± 1.0	8.0±3.2
	PLA/Bleached Flax	60.0±0.2	40 ± 0.2	6.2±3.1

Table S4 - Fibre and polymer weight percentage and porosity percentage of composite filaments produced using emulsion impregnation and consolidation method.

Formulation	Composite Filament	Wt % of Reinforcement	Wt % of Polymer	Porosity
**Emulsion x1**	PLA/Viscose	48.0±1.4	52.0±1.4	10.3±3.0
	PLA/Bleached Flax	69.5±0.3	30.5±0.3	17.0±2.0
**Emulsionx2**	PLA/Viscose	41.2±1.3	58.8±1.3	7.4±1.6
	PLA/Bleached Flax	68.1±1.1	31.9±1.1	8.9±3.0
**Emulsionx3**	PLA/Viscose	40.0±0.2	60.0±0.2	4.0±2.5
	PLA/Bleached Flax	58.0±0.4	42.0±0.2	9.3±5.7
**Emulsionx1 (tandem)**	PLA/Viscose	42.1±0.8	57.9±0.8	5.1±2.1
	PLA/Bleached Flax	69.7±1.3	30.3±1.3	9.8±4.3
**Emulsionx2 (tandem)**	PLA/Viscose	40.0±1.4	60.0±1.4	4.7±2.3
	PLA/Bleached Flax	41.0±0.5	59.0±0.5	7.8±4.0

6 - Tensile test data of single yarn reinforced filaments

Table S5 - Tensile properties of composite filaments produced from solution impregnation and consolidation method.

Formulation	Composite Filament	Wt % Reinfor-cement	Tensile Strength (MPa)	Young's Modulus (GPa)	Strain at break (%)
**10wt%x1**	PLA/Viscose	76.8±0.2	230.7±16.1	6.7±0.4	10.7±1.6
	PLA/Bleached Flax	77.80±0.2	215.9±12.8	7.3±3.2	4.2±1.4
**10wt%x2**	PLA/Viscose	63.2±0.9	235.3±10.1	7.2±1.7	15.6±3.2
	PLA/Bleached Flax	72.2±1.2	219.6±28.1	8.5±2.1	4.1±0.4
**7wt%x2**	PLA/Viscose	65.5±2.0	240.8±24.6	7.9±1.5	15.4±2.5
	PLA/Bleached Flax	74.80±1.4	293.1±28.0	10.6±1.2	4.1±0.6
**7wt%x3**	PLA/Viscose	63.1±0.6	233.8±16.2	7.5±1.2	12.4±2.3
	PLA/Bleached Flax	64.3±0.1	356.1±6.8	11.6±1.8	4.2±0.4
**7wt%x1 (tandem)**	PLA/Viscose	70.8±0.6	245.1±7.6	6.9±1.5	12.9±1.8
	PLA/Bleached Flax	69.2±0.2	302.7±11.3	10.4±2.8	3.8±0.3
**7wt%x2 (tandem)**	PLA/Viscose	64.3±1.0	233.0±8.1	7.3±1.0	14.8±1.2
	PLA/Bleached Flax	60.0±0.2	326.1±15.5	17.6±0.8	3.2±0.3

Table S6 - Tensile properties of composite filaments produced using emulsion impregnation and consolidation method.

Formulation	Composite Filament	Wt % Reinfo-rcement	Tensile Strength (MPa)	Young's Modulus (GPa)	Strain at break (%)
**Emulsion x1**	PLA/Viscose	48±1.4	246.5±17.4	7.5±1.4	12.5±4.8
	PLA/Bleached Flax	69.5±0.3	215.9±16.8	6.4±1.9	4.4±0.9
**Emulsionx2**	PLA/Viscose	41.2±1.3	254.7±15.3	9.1±0.4	14.7±2.1
	PLA/Bleached Flax	68.1±1.1	227.8±15.2	7.0±1.9	3.8±0.6
**Emulsionx3**	PLA/Viscose	40.0±0.2	250.8±15.4	8.1±1.2	11.7±1.9
	PLA/Bleached Flax	58±0.4	291.6±19.8	12.3±1.3	3.1±0.2
**Emulsionx1 (tandem)**	PLA/Viscose	42.1±0.8	247.2±18.8	8.4±0.7	15.2±1.4
	PLA/Bleached Flax	69.7±1.3	251.1±15.1	9.2±1.9	3.1±0.5
**Emulsionx2 (tandem)**	PLA/Viscose	40.00±1.4	241.8±19.6	8.1±1.6	14.3±4.6
	PLA/Bleached Flax	41±0.5	296.2±22.1	12.6±1.1	3.2±0.3

7 - Polymer and porosity percentage of multiple consolidated filaments and 3D printing filaments

Table S7 - Fibre and polymer weight percentage and porosity percentage of multiple consolidated filaments.

Impregnation Type	Composite Filament	Fibre wt%	Polymer wt%	Porosity (%)
**Solution impregnation**	PLA/Viscose	71.4±2.3	28.6±2.3	9.5±2.7
	PLA/Bleached Flax	70.3±5.9	29.7±5.9	16.7±1.4
**Emulsion impregnation**	PLA/Viscose	62.4±2.9	37.6±2.9	6.0±2.2
	PLA/Bleached Flax	54.2±2.0	45.8±2.0	10.0±1.5

Table S8 - Fibre and polymer weight percentage and porosity percentage of melt impregnated 3D printing filaments.

Impregnation type	Composite Filament	Fibre wt%	Polymer wt%	Porosity (%)
**Solution impregnation**	PLA/Viscose	33.4±1.3	66.6±1.3	4.4±1.8
	PLA/Bleached Flax	27.8±2.1	72.2±2.1	5.2±0.6
**Emulsion impregnation**	PLA/Viscose	32.8±0.9	67.2±0.9	5.0±2.1
	PLA/Bleached Flax	26.4±2.8	73.6±2.8	5.5±1.9

8 - Tensile properties of multiple consolidated filaments and 3D printing filaments

Table S9 - Tensile properties of multiple consolidated filaments.

Impregnation Type	Material	Fibre wt%	Tensile Strength (MPa)	Young's Modulus (GPa)	Strain at break (%)
**Solution impregnation**	PLA/Viscose	71.4±2.3	251.2±15.2	8.8±0.8	21.2±3.9
	PLA/Bleached Flax	70.3±5.9	305.7±23.8	15.5±2.2	4.0±0.5
**Emulsion impregnation**	PLA/Viscose	62.4±2.9	245.3±13	7.5±0.5	25.8±4.5
	PLA/Bleached Flax	54.2±2.0	215.9±12.8	9.8±0.9	3.3±0.4

Table S10 -Tensile properties of 3D printing filaments.

Impregnation Type	Material	Fibre wt%	Tensile Strength (MPa)	Young's Modulus (GPa)	Strain at break (%)
**Solution impregnation**	PLA/Viscose	33.4±1.3	201.8±19.6	6.8±3.6	17.5±2.8
	PLA/Bleached flax	27.8±2.1	180.9±7.4	9.6±5.3	3.0±0.2
**Emulsion impregnation**	PLA/Viscose	32.8±0.9	190.2±15.9	7.4±1.7	22.1±3.9
	PLA/Bleached flax	26.4±2.8	154.7±16	9.2±5.5	3.4±0.9

9 – DSC of 3D printing filaments and 3D printed samples.

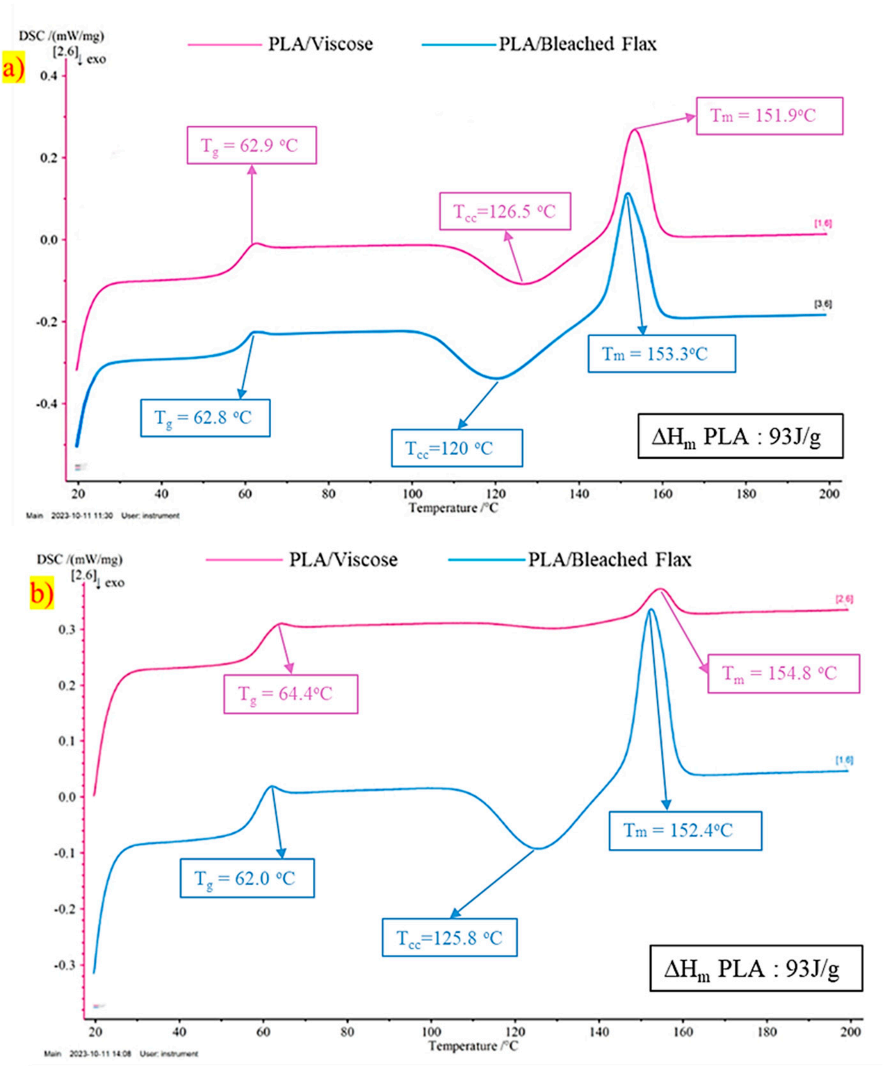

Figure S18 - DSC thermograms of 3D printing composite filaments a) Solution impregnation b) Emulsion impregnation.

Table 1 Summary of DSC for 3D printing composite filaments

Impregnation Type	Composite Filament	T_g (°C)	T_m (°C)	T_{cc} (°C)	X_c (%)
Solution Impregnation	PLA/Viscose	62.9	151.9	126.5	24.3
	PLA/Bleached Flax	62.8	153.3	120.0	19.2
Emulsion Impregnation	PLA/Viscose	64.4	154.8	-	-
	PLA/Bleached Flax	62.0	152.4	125.8	21.2

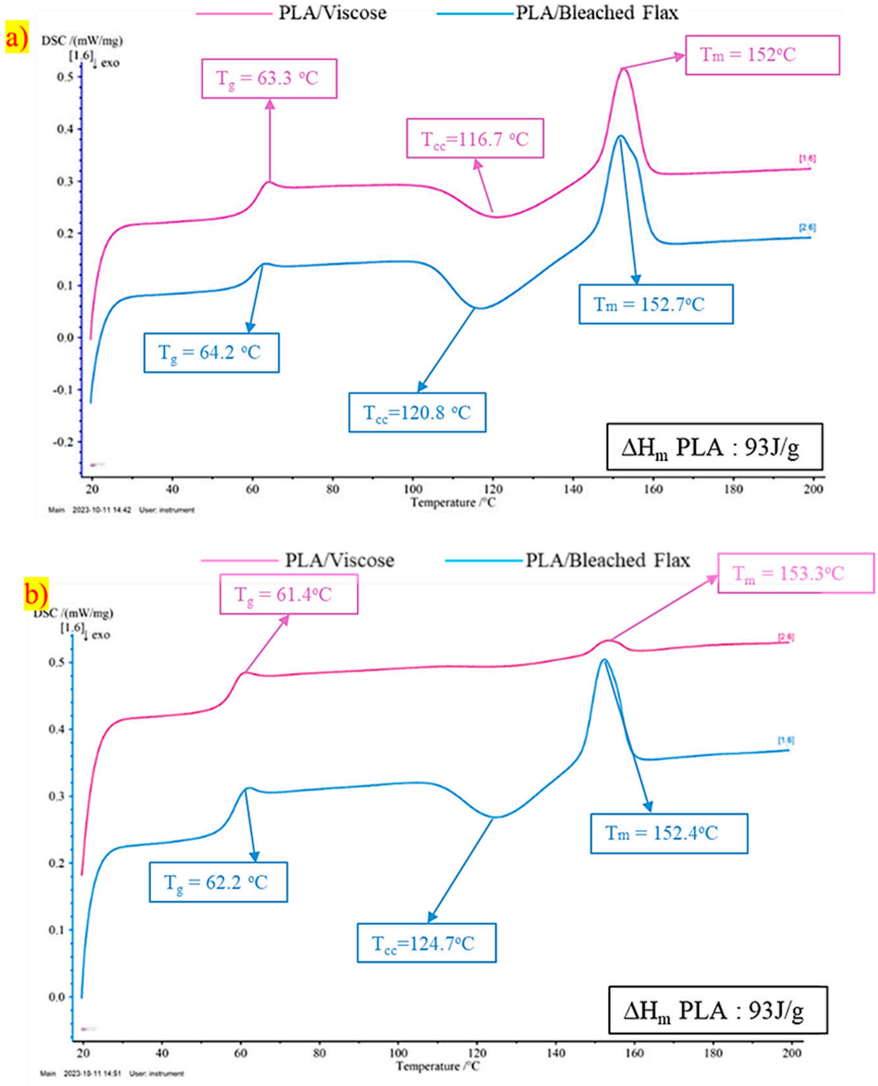

Figure S19 - DSC thermograms of FDM 3D printed composites a) Solution impregnation b) Emulsion impregnation

Table S12 - Summary of DSC of 3D printed composites.

Impregnation Type	3DP Composite	T_g (°C)	T_m (°C)	T_{cc} (°C)	X_c (%)
**Solution Impregnation**	PLA/Viscose	63.3	152	116.7	19.7
	PLA/Bleached Flax	64.2	152.7	120.8	14.2
**Emulsion Impregnation**	PLA/Viscose	61.4	153.3	-	-
	PLA/Bleached Flax	62.2	152.4	124.7	10.5

References

[1] Wang S, Lu A, Zhang L. Recent advances in regenerated cellulose materials. Prog Polym Sci 2016;53:169–206. https://doi.org/10.1016/j.progpolymsci.2015.07.003.

[2] Thomason JL, Carruthers J, Kelly J, Johnson G. Fibre cross-section determination and variability in sisal and flax and its effects on fibre performance characterisation. Compos Sci Technol 2011;71:1008–15. https://doi.org/https://doi.org/10.1016/j.compscitech.2011.03.007.

[3] Bunsell AR, Joannès S, Marcellan A. 2 - Testing and characterization of fibers. In: Bunsell AR, editor. Handbook of Properties of Textile and Technical Fibres (Second Edition). Second Edi, Woodhead Publishing; 2018, p. 21–55. https://doi.org/https://doi.org/10.1016/B978-0-08-101272-7.00002-X.

[4] ASTM D3379-75; Standard Test Method for Tensile Strength and Young’s Modulus for High-Modulus Single-Filament Materials. ASTM International: West Conshohocken, PA, USA, 2000

[5] Pilla S, Gong S, O’Neill E, Rowell RM, Krzysik AM. Polylactide-pine wood flour composites. Polymer Engineering & Science 2008;48:578–87. https://doi.org/10.1002/pen.20971.
